# Characterizing the electrophysiological abnormalities in visually reviewed normal EEGs of drug-resistant focal epilepsy patients

**DOI:** 10.1093/braincomms/fcab102

**Published:** 2021-05-14

**Authors:** Yogatheesan Varatharajah, Brent Berry, Boney Joseph, Irena Balzekas, Tal Pal Attia, Vaclav Kremen, Benjamin Brinkmann, Ravishankar Iyer, Gregory Worrell

**Affiliations:** 1 Department of Bioengineering, University of Illinois, Urbana, IL 61801, USA; 2 Mayo Systems Electrophysiology Laboratory, Department of Neurology, Mayo Clinic, Rochester, MN 55905, USA; 3 Electrical and Computer Engineering, University of Illinois, Urbana, IL 61801, USA; 4 Czech Institute of Informatics, Robotics and Cybernetics, Czech Technical University in Prague, 160 00 Prague 6, Czech Republic

**Keywords:** routine EEG, diagnosis of epilepsy, alpha rhythm, visual EEG review, spectral analysis

## Abstract

Routine scalp EEG is essential in the clinical diagnosis and management of epilepsy. However, a normal scalp EEG (based on expert visual review) recorded from a patient with epilepsy can cause delays in diagnosis and clinical care delivery. Here, we investigated whether normal EEGs might contain subtle electrophysiological clues of epilepsy. Specifically, we investigated (i) whether there are indicators of abnormal brain electrophysiology in normal EEGs of epilepsy patients, and (ii) whether such abnormalities are modulated by the side of the brain generating seizures in focal epilepsy. We analysed awake scalp EEG recordings of age-matched groups of 144 healthy individuals and 48 individuals with drug-resistant focal epilepsy who had normal scalp EEGs. After preprocessing, using a bipolar montage of eight channels, we extracted the fraction of spectral power in the alpha band (8–13 Hz) relative to a wide band of 0.5–40 Hz within 10-s windows. We analysed the extracted features for (i) the extent to which people with drug-resistant focal epilepsy differed from healthy subjects, and (ii) whether differences within the drug-resistant focal epilepsy patients were related to the hemisphere generating seizures. We then used those differences to classify whether an EEG is likely to have been recorded from a person with drug-resistant focal epilepsy, and if so, the epileptogenic hemisphere. Furthermore, we tested the significance of these differences while controlling for confounders, such as acquisition system, age and medications. We found that the fraction of alpha power is generally reduced (i) in drug-resistant focal epilepsy compared to healthy controls, and (ii) in right-handed drug-resistant focal epilepsy subjects with left hemispheric seizures compared to those with right hemispheric seizures, and that the differences are most prominent in the frontal and temporal regions. The fraction of alpha power yielded area under curve values of 0.83 in distinguishing drug-resistant focal epilepsy from healthy and 0.77 in identifying the epileptic hemisphere in drug-resistant focal epilepsy patients. Furthermore, our results suggest that the differences in alpha power are greater when compared with differences attributable to acquisition system differences, age and medications. Our findings support that EEG-based measures of normal brain function, such as the normalized spectral power of alpha activity, may help identify patients with epilepsy even when an EEG does not contain any epileptiform activity, recorded seizures or other abnormalities. Although alpha abnormalities are unlikely to be disease-specific, we propose that such abnormalities may provide a higher pre-test probability for epilepsy when an individual being screened for epilepsy has a normal EEG on visual assessment.

## Teaser

Varatharajah et al. report that subtle abnormalities in EEG measures such as the spectral power of alpha activity may help identify (i) patients with epilepsy, and (ii) the seizure generating hemisphere in focal epilepsy, even when a routine scalp EEG is free of any epileptiform activity or other visible abnormalities.

## Graphical Abstract

**Graphical Abstract:**
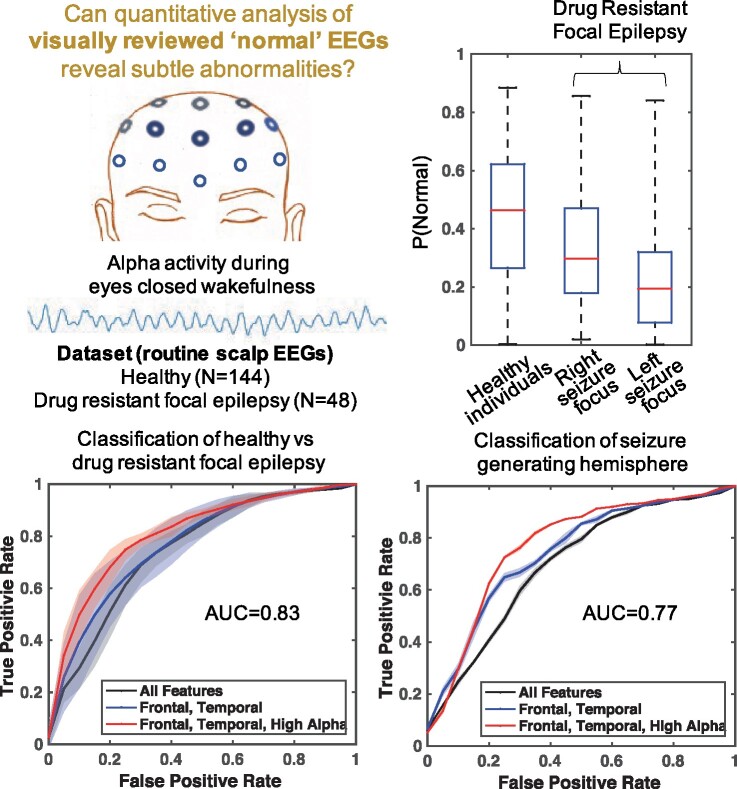

## Introduction

Epilepsy is a neurological disease characterized by recurrent, unprovoked seizures and affects 1% of the global population.^1^ Epileptologists assess the potential for epilepsy and related conditions by visually identifying abnormal activity (also known as epileptiform activity) in a short scalp EEG recording session (∼20–60 min). A positive screen, the presence of abnormal epileptiform EEG transients, is typically followed by the initiation of anti-seizure medication (ASM) and further evaluation for an aetiology. However, this initial assessment is not always sensitive enough, as epileptiform activity may not be recorded in a short EEG session. Unfortunately, such scenarios are very common in clinical settings,^2^ and some patients ultimately found to have drug-resistant epilepsy (DRE) have normal EEGs on expert visual review (i.e. the EEGs did not contain any epileptiform activity).^3^^,^^4^ The inability to find evidence for epilepsy at the earliest possible time can cause delays in delivering appropriate and early clinical care.^5^ Even after ASMs are initiated, approximately one-third of people will not completely respond and continue to have seizures despite multiple different medication trials, i.e. DRE. Each medication trial can take months, thus putting the patient at continued risk for seizure-related injuries and comorbidities.^6^ Furthermore, in patients ultimately diagnosed with DRE, a more comprehensive evaluation is indicated to determine if they are candidates for non-pharmacological therapies, e.g. surgery and electrical stimulation. Thus, a more rapid diagnosis of epilepsy, and in particular DRE, is needed. In this study, we evaluate the hypothesis that even short EEG recordings might contain subtle electrophysiological abnormalities that can indicate the possibility of epilepsy even when recognizable epileptiform activity is absent. If confirmed, this could improve the diagnostic yield of routine EEG and facilitate more sensitive, objective, and earlier diagnosis and treatment of epilepsy.

EEG plays a crucial role in diagnosing epilepsy.^7^ Electrodes are attached to an individual’s scalp to record brain electrical activity. In people with epilepsy, it is common to see transient voltage disruptions to the normal pattern of brain waves, even in the interictal recording when a patient is not having a seizure.^8^ The most common abnormalities in brain activity associated with epilepsy are interictal spikes and sharp waves.^9^ Interictal spikes and sharp waves represent the summated excitatory and inhibitory post-synaptic potentials of a large population of neurons^10^ and have similar underlying physiological causes. The difference in their appearances reflects the rapidity of neuronal synchronization and the way in which the epileptiform discharge spreads over the cortex. In addition to providing evidence for epilepsy, the spatial distribution of these events can identify epilepsy as having generalized or focal origins. In focal epilepsy, the distribution of interictal spikes and sharp waves can help spatially map epileptogenic brain regions.^11^ However, these abnormalities may not be observed in short EEG recordings for multiple reasons, e.g. they may be very infrequent and not captured on routine ∼30-min recording, they may originate from deeper brain structures like cingulate, hippocampus, etc., they are activated only during sleep that was not recorded, or they involve an insufficient amount of cortex to be measurable on the scalp.^12^

Pathologic changes, such as neuronal loss and gliosis, are common in chronic epilepsy, though the same neuronal-glial circuits underlying seizure generation may subserve normal brain functions.^13^^,^^14^ The cellular changes associated with epilepsy may be expected to cause subtle declines in EEG-based measures of normal brain function. The presence of such abnormalities can provide a higher pre-test probability for epilepsy when an individual is first screened for epilepsy and could therefore warrant additional testing when their EEG does not contain epileptiform activity, e.g. prolonged EEG recording that includes sleep. Several previous studies have analysed EEG biomarkers of normal brain function in the EEG segments of epilepsy patients without interictal abnormalities and reported results supporting the aforementioned hypothesis. The EEG measures investigated include spectral connectivity measures based on phase-locking factor,^15–17^ spectral connectivity based on weighted partial directed coherence,^18^ and local and global synchrony measures based on network modelling of EEG activity.^19^ Furthermore, the alpha rhythm observed on EEG during eyes-closed wakefulness is considered another potential biomarker of normal brain function in adults, and its frequency and power decrease with age.^20^ It is theorized to arise through cortico-thalamic interactions and to reflect processes that subserve a vast range of cognitive processes, including attention and memory.^21^ Alterations in the alpha rhythm have been observed in many neurological diseases, including epilepsy, where it typically slows in frequency and loses its characteristic anterior-to-posterior gradient proportionally with clinical severity.^22^ Although alpha-rhythm-related abnormalities are well known in epilepsy, including associations with poor seizure control,^23^^,^^24^ they are not included in diagnostic criteria because they lack specificity to any neurological disease.^25^^,^^26^ In addition, the analysis of alpha rhythm in the EEGs of epilepsy patients is further complicated by its changes related to ageing^20^ and antiepileptic, antidepressant and antipsychotic medications.^27–29^ Despite the considerable literature focussing on normal EEG segments, there is very little evidence supporting whether these findings translate to EEGs that are entirely free of epileptiform abnormalities or whether quantitative analysis of these normal records could be clinically useful. Furthermore, whether the previously established relationships are resilient to changes induced by medications and ageing and differences in EEG recording conditions remains unclear.

In this study, we focus on spectral characteristics of the alpha rhythm and investigate whether we can identify subtle abnormalities in routine EEGs that were visually classified as normal by practicing epileptologists, particularly in DRE. Additionally, there is evidence that people with epilepsy who have seizures originating from their dominant hemisphere can experience relatively more disruptions in their normal brain function compared to those with seizure foci in their non-dominant hemispheres.^30^ Therefore, we further hypothesize that in addition to indicating the potential for epilepsy, alpha rhythm abnormalities can also help lateralize the seizure focus in focal epilepsy. To test those hypotheses, we analysed the scalp EEG recordings of healthy individuals age-matched to people with epilepsy, specifically drug-resistant focal epilepsy (DRFE), who went through clinical evaluations at the Mayo Clinic. The EEGs of DRFE individuals were classified as normal based on expert visual review performed by board-certified epileptologists. We preprocessed the EEGs to remove artefacts and performed spectral analysis to extract EEG features representing the fraction of spectral powers contained within the alpha band across the four major brain regions. We then used the extracted features to analyse (i) the extent to which DRFE patients deviated from healthy, and (ii) whether there are differences within the DRFE patients based on the hemisphere generating seizures. Furthermore, we analysed whether these differences are significant when compared with confounders such as the individual’s age, acquisition system differences, and antiepileptic drugs.

## Materials and methods

Our primary analyses utilized the scalp EEG data of 144 healthy individuals age-matched to 48 patients with DRFE. We also utilized the normal scalp EEG data of 104 patients with psychogenic non-epileptic seizures (PNES) to analyse the effect of different EEG acquisition systems on our findings. We obtained the data on healthy individuals from the publicly available LEMON dataset^31^ and the data of DRFE and PNES patients from clinical records at the Mayo Clinic, Rochester, MN, USA.

### Data from healthy individuals

The LEMON dataset consisted of scalp EEG recordings from 203 healthy individuals (median age 39, age-range 20–77, 82 females).^31^ The participants were stratified between *young* and *old* groups with age ranges 20–35 and 59–77, respectively. The EEGs were recorded using the BrainAmp MR plus recording system with the ActiCAP electrodes (both from Brain Products GmbH, Gilching, Germany), including 62 channels according to the standard 10–10 localization system.^32^ The data were originally recorded at a sampling rate of 2500 Hz and then downsampled to 250 Hz, and each EEG session comprised eight eyes-closed (EC) and eight eyes-open (EO) segments, each 60 s long. We rejected the channels that were determined as outlier channels by the investigators of the LEMON study.^31^ As a result, we excluded the data of 59 individuals from our study because some channels required for our analyses were either not available or deemed outlier channels by the LEMON investigators.

### Data from people with DRFE and PNES

We obtained scalp-EEG recordings from 48 individuals with DRFE (median age 39, age-range 18–66, 25 females) and 104 individuals with PNES (median age 30.5, age-range 18–62, 60 females) that were performed as part of their clinical evaluations. Our study was approved by the Mayo Clinic Institutional Review Board, and patients provided informed consent. The EEGs were recorded using the XLTEK EMU40EX headbox (from Natus Medical Incorporated, Oakville, Ontario, Canada) with the WaveGuard Original EEG cap (from Ant Neuro GmbH, Berlin, Germany) including 31 channels according to the extended 10–20 localization system^33^ at a sampling rate of 256 Hz. The EEGs were visually reviewed by board-certified epileptologists and classified as normal. We selected EC segments based on the annotations made by EEG technologists during the clinical video-EEG review.

### Main experiments

Our first analysis focussed on differences between epilepsy patients and healthy individuals. We utilized the data of all 144 healthy individuals from the LEMON study and 48 Mayo patients for this analysis. Our second analysis focussed on differences based on the seizure-generating hemisphere of the brain in epilepsy patients. In addition to the EEG features, we utilized handedness (right or left) to determine the dominant hemisphere. Of the 48 epilepsy patients, 43 were right-handed, and 5 were left-handed. Furthermore, although there is evidence that the dominant hemisphere of right-handed individuals is generally deterministic (i.e. the left side), the dominant hemisphere of left-handed individuals is non-deterministic (can be the left or the right side).^34^ Therefore, we excluded the (i) left-handed patients because of insufficient sample size and the non-deterministic nature of their dominant hemisphere, and (ii) one right-handed patient who had a central midline (non-lateralized) seizure onset from this analysis. As a result, the second analysis utilized the EEG data of 42 right-handed DRFE patients (28 patients with seizure focus on the left side and 14 patients with seizure focus on the right side).

### Analysis of confounders

We compared the effect of age against the group differences between healthy and DRFE individuals. We divided the healthy population into young (97 individuals between ages 20 and 35) and old (47 individuals between ages 59 and 77) populations and compared them with the DRFE patients. To analyse whether the group differences between healthy and DRFE individuals were distinct from acquisition system differences, we performed a three-way comparison between all healthy, PNES and DRFE individuals. Here, the EEGs of PNES and DRFE patients were recorded using the same acquisition system. Because both the DRFE and PNES populations were heterogeneous with respect to medications, as an extension of the previous comparison, we performed a second comparison by limiting the two patient populations to those taking ASMs only (27 PNES patients and 43 DRFE patients). We then compared the effects of ASMs in three ways: (i) differences between DRFE patients who were not taking any ASMs (5 patients) compared to those who did (43 patients); (ii) differences between PNES patients who were not taking any ASMs (44 patients) compared to those who did (27 patients); and (iii) differences between DRFE patients who were taking either levetiracetam (25 patients) or lamotrigine (19 patients), the most common ASMs in this study. Note that we did not include any patients who were receiving antipsychotics or selective serotonin reuptake inhibitor drugs and patients with other significant comorbidities in the aforementioned groups comparing the effects of ASMs. See Supplementary Tables 1 and 2 for more details. Finally, we analysed the differences in arousal states between healthy and DRFE individuals. It is plausible that DRFE patients are in a more drowsy state at the time of EEG recording because of medication effects, previous seizures, and sleep deprivation. To analyse this effect, we utilized the rate of eye blinks as a surrogate for drowsiness^35^^,^^36^ and compared the number of eye blinks per minute in the eyes open EEG segments of healthy and DRFE individuals.

### EEG preprocessing

Further preprocessing was done in EEGLAB for MATLAB.^37^ First, the EEGs were bandpass-filtered within 1–45 Hz (8th order, Butterworth filter). Next, an independent component analysis was performed, and components reflecting eye movement, eye blink or heartbeat-related artefacts were removed, and bad channels were rejected, all according to the widely recognized Makoto’s EEG preprocessing pipeline.^38^ The retained independent components were back-projected to sensor space for further analysis.

### Counting eye blinks

Eye blinks were detected and counted using an automated tool available as part of the EEGLAB software.^39^ Eyeblink detection is performed at two levels of specificity: (i) all potential eye blinks chosen as segments with signal amplitude greater than 1.5 standard deviations above the overall mean amplitude and durations longer than 50 ms, and (ii) specific blinks within all potential blinks that have significantly correlated upstrokes and downstrokes (*R*^2^ > 0.90). More details on the tool’s implementation can be found in the original manuscript.

### Selection of EEG channels

Because the EEG data of healthy individuals were recorded using a 62-channel 10–10 system, we selected a subset of channels that matched the 10–20 system used to record the EEG data from epilepsy patients. Within the EEG data of selected channels from the healthy and patient populations, we selected four bipolar pairs of electrodes from each hemisphere, producing eight channels of EEG data for each participant. Table 1 shows the electrodes that were used to form the bipolar montage representing each major brain region and hemisphere.

**Table 1:** Channels used to form the bipolar montage representing each major brain region and hemisphere

Left hemisphere	Right hemisphere
F7–F3	F8–F4
T7–C3	T8–C4
P7–P3	P8–P4
O1–P3	O2–P4

### Extracting spectral features

**Figure 1:**
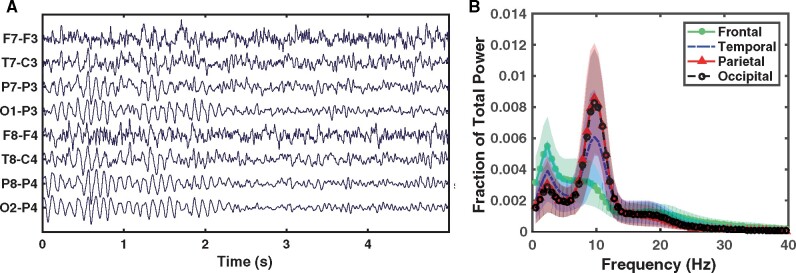
**Extraction of spectral features.** (**A**) illustrates a sample EC segment (length: 5-s) of a healthy participant’s EEG. (**B**) illustrates normalized power spectrums of EC segments, where the solid line indicates average values calculated across all the healthy participants and the shaded areas indicate 95% confidence intervals.

We first normalized (*z*-scored) each channel separately within each segment and divided each segment into 10-s non-overlapping windows (Fig. 1A, only 5 s long EEGs are showed for visual clarity). Note that the number of 10-s windows was different for each participant in the DRFE and PNES populations. We then computed the power spectrum for each window using the multitaper spectral estimation methods implemented in the Chronux toolbox.^40^ In order to eliminate the subject-specific differences in total signal power, we also normalized all the calculated power values by using the total power of the signal within 0.5–40 Hz (Fig. 1B). We then separately aggregated the normalized power within two frequency bands: low-alpha (7.5–10.5 Hz) and high-alpha (10.5–13.5 Hz); such a division of the alpha band allows the detection of slowing (more power in low-alpha than in high-alpha) as well as the overall reduction in alpha power. As a result, each calculated feature was a fraction of the total power within one of the alpha bands, and each 10-s window produced 16 features (8 channels × 2 frequency bands). Furthermore, each participant's EEG data produced $N_{EC}\times 16$ features, where $N_{EC}$ is the number of EC windows. The healthy data consisted of an average of 47 EC windows (min = 42, max = 48), the DRFE patient data consisted of an average of 13 EC windows (min = 4, max = 69), and the PNES patient data consisted of an average of 11 EC windows (min = 5, max = 22).

### Characterizing normal brain activity

**Figure 2:**
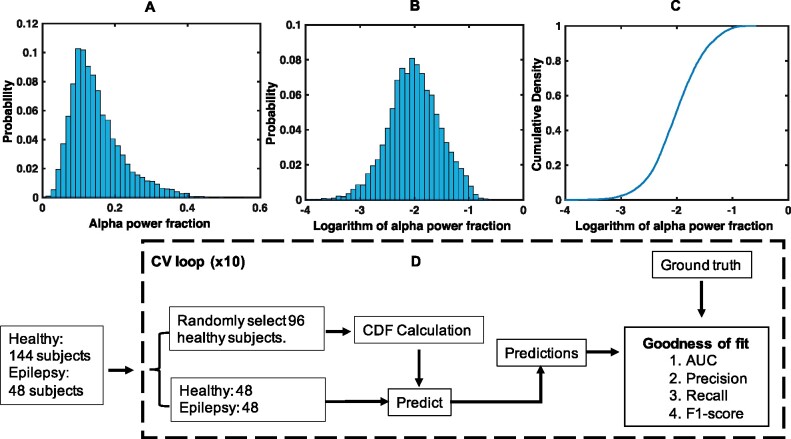
**Characterizing normal brain function & classification framework.** (**A**) Histogram of low-alpha (7.5–10.5 Hz) power fraction in (F7-F3) in the EC windows of healthy individuals. (**B**) and (**C**) illustrate the histogram and the cumulative density of the log transformation of the same features, respectively. (**D**) A random sample of 96 healthy participants is selected for characterizing normal brain function. The data of the rest (48 healthy and 48 DRFE) are utilized to evaluate the classification potential. Cumulative density functions of the 16 features representing alpha power fractions in the EC segments are computed. Window-level probability-of-normality estimates were aggregated to obtain participant-level probabilities. The goodness-of-fit metrics were computed by comparing participant level probabilities with ground truth, separately for classifying (i) healthy versus epilepsy, and (ii) hemisphere with seizure focus. This procedure was repeated ten times to estimate average metrics and standard deviations.

We performed a log transformation of the features and used the cumulative density function (CDF) of the log-transformed features in the healthy population to characterize normal brain function. An example of this is pictorially illustrated in Fig. 2A–C. Suppose that the CDF of a single log-transformed feature $x_{k}$ (k∈{1,…,16}) is the feature number in the healthy population (considering all participants) is denoted by $F(x_{k})$, where $x_{k}\in [-\infty ,0]$ and $F\left(x_{k}\right)\in [0,1]$. We hypothesize that the samples representing abnormal brain function will fall near the lower limit of $x_{k}$, suggesting that the likelihood of those samples coming from a population with normal brain function, which we refer to as the *probability-of-normality*, is low. Therefore, the probability-of-normality for such samples will be close to zero, while the probability-of-normality for samples that are near the upper limit of $x_{k}$ will be close to one. With this intuition, we use the CDF of $x_{k}$ to estimate the probability-of-normality for a new sample $x_{k}^{\prime }$, i.e. $P(x_{k}^{\prime }\in \mathrm{Normal})=F(x_{k}^{\prime })$, as the CDF satisfies the requirements above. However, note that this estimation of probability-of-normality is based on a single feature $x_{k}$. Although these single-feature-based estimates can be directly compared across individuals, an approach to combine those differences within each individual is necessary to develop an individual-level classification scheme and evaluate the clinical value of those differences. In the following, we describe an approach to combine the probability-of-normality estimates within each individual.

### Calculating probability-of-normality for an EC window

To combine the values of all 16 features in an EC window, we assume that the individual estimations can be independently combined. By applying the independence assumption, we now derive a combined probability-of-normality, as shown below.

(1) $$P\left(x^{'}\in \mathrm{Normal}\right)=\sqrt[{16}]{\prod_{k=1}^{16}P(x_{k}^{'}\in \mathrm{Normal})}=e^{\frac{\sum_{k=1}^{16}\log\left(P\left(x_{k}^{'}\in \mathrm{Normal}\right)\right)}{16}}$$

Note that we use the geometric mean of the product of the individual feature-based estimates as the combined estimate because the multiplication of 16 fractional numbers will produce a very small probability value. Furthermore, we calculated $P\left(x^{\prime }\in \mathrm{Normal}\right)$ described in (1) in the log domain to avoid numerical instability. This derivation provides a single probability value $P\left(x^{\prime }\in \mathrm{Normal}\right)$ for each EC window. Using the same approach, we can calculate window-level probability-of-normality values for all EC windows of all participants.

### Individual-level probability estimation

To estimate whether a participant’s EEG is *normal*, we used a maximum likelihood estimation based on the window-level probability-of-normality values. We model the window-level estimates of a participant $P_{i}$ as independent observations made from a Bernoulli trial with an unknown probability $\pi_{i}$, where $\pi_{i}$ is the probability-of-normality for participant $P_{i}$, i.e. $\pi_{i}=P(P_{i}\in \mathrm{Normal})$. Suppose that we use $x^{\prime }\left(i,n\right)$ to denote the $n^{th}$ window of participant i, Y(i,n) to denote $P\left(x^{\prime }(i,n)\in \mathrm{Normal}\right)$, and the total number of windows are denoted using N(i). Then, an estimate of $\pi_{i}$ that maximizes the likelihood function $\prod_{n=1}^{N\left(i\right)}\pi_{i}^{Y\left(i,n\right)}{\left(1-\pi_{i}\right)}^{\left(1-Y\left(i,n\right)\right)}$ is given as the following.

(2) $$\hat{\pi_{i}}=\frac{1}{N(i)}\sum_{n=1}^{N(i)}Y(i,n)$$

### Computing differences between CDFs

In order to characterize the group differences based on the CDFs, we utilized the Wasserstein distance (or earth mover’s distance) metric.^41^

### Visualizing group differences using boxplots

We performed the following operations to generate boxplots. First, we computed the CDFs of the 16 features (i.e. the log-transformed alpha-power fractions of an EC window) using the data of all 144 healthy individuals. Then, we calculated the window-level probability-of-normality estimates for the groups of individuals we were interested in comparing, using the computed CDFs (see previous sections for more information). Those values were then divided among the comparison groups to generate the boxplots. For reference, we also plot the probability-of-normality estimates for the same healthy individuals whose CDFs are used to generate those estimates.

### Statistical analysis

In order to test the statistical significance of the differences between the two distributions, we used the Wilcoxon rank-sum test.^42^ Furthermore, we performed multiple comparisons between different stratifications in the study population using Tukey’s honestly significant difference procedure.^43^

### Headplots

Headplots illustrating the spatial distributions of features of interest were plotted using the headplot() function in the EEGLAB toolbox.^37^ The headplots are generated using a spherically splined field map of the feature of interest on a semi-realistic head model.

### Classification framework

Figure 2D illustrates the approach we utilized for classifying healthy and DRFE patients using ten-fold cross-validation. During each cross-validation, we selected a random sample of 96 healthy participants to generate the feature-specific CDFs characterizing normal brain function (i.e. training set). We used the data of the rest of the participants (48 healthy and 48 DRFE) to evaluate the classification potential of our approach (i.e. testing set). This scheme ensured that the training and testing datasets consisted of two disjoint sets of participants and that the testing set is class-balanced. First, we computed the CDFs of the 16 features representing alpha power fractions in the EC windows using the healthy sample in the training set. Then, we computed the window-level probability-of-normality values for each EC window in the testing set. Then, we aggregated the window-level values to obtain individual-level values as described previously. By comparing individual-level probabilities with ground truth, we computed goodness-of-fit metrics for the classification task, separately for classifying (i) healthy versus epilepsy, and (ii) hemisphere with seizure focus.

### Performance evaluation

We first plotted receiver operating characteristic (ROC) curves and calculated the area under the ROC curve (AUC) to compare model performances. An optimal threshold on the ROC curve was selected using the convex hull method.^44^ That threshold was used to calculate precision, recall, and F1-score. The classification procedure was repeated ten times to calculate the mean and standard deviations of the metrics. In addition, because frontal and temporal lobe epilepsy are the most common forms of focal epilepsy, we performed the classification approach using the features from those two regions alone and compared the results with those obtained from the approach using features extracted from all regions.

### Data availability

The data of healthy controls is already publicly available. The deidentified spectral features of healthy individuals and patients and the software used to perform statistical analyses are available at https://gitlab.engr.illinois.edu/varatha2/epilepsy_alpha_rhythm.git (last accessed: May 26, 2021).

## Results

### Characterizing normal brain function in the healthy population

**Figure 3:**
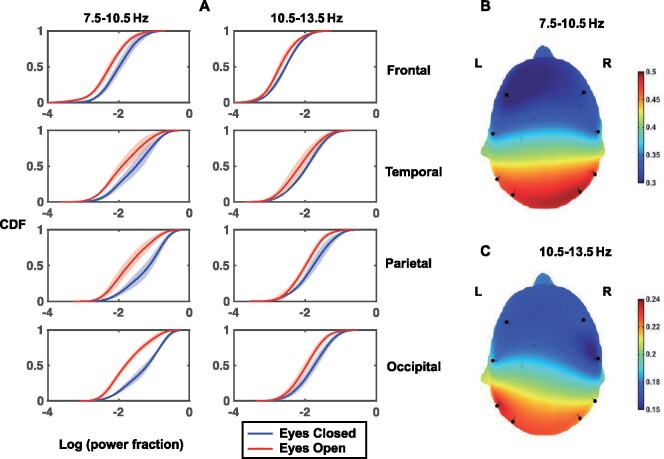
**Characterizing normal brain function in the healthy population.** (**A**) Cumulative density functions of log-transformed spectral power fractions in low-alpha (7.5–10.5 Hz) and high-alpha (10.5–13.5 Hz) bands, grouped based on eyes-closed and eyes-open conditions. Solid lines indicate average values across 10-s windows, and shaded areas indicate 95% confidence intervals. Note that the window-level features were averaged between the left and right hemispheres to generate the CDF plots. (**B**) and (**C**) are head plots illustrating the location-specific Wasserstein distances between the CDFs of EC and EO windows in low-alpha (7.5–10.5 Hz) and high-alpha (10.5–13.5 Hz) bands, respectively. The pins indicate the approximate locations of the channels.

We used the frequency domain features based on the alpha rhythm power to characterize normal brain function. Figure 3A illustrates the CDFs of log-transformed alpha-power fractions in the healthy population in EC and EO windows. The figure also highlights the differences between the four major brain regions with respect to the same features, where the CDF of each brain region was generated by taking the average of the CDFs of the respective regions in the left and right hemispheres. Our observations agree with the commonly known characteristics of the alpha rhythm, i.e. (i) it is posteriorly dominant, and (ii) its presence is amplified when the eyes are closed.^25^ In addition, Fig. 3B and C shows the spatial patterns of the differences between EC and EO conditions in the two alpha bands, based on Wasserstein distances between the CDFs. We find that the differences are greater in the posterior regions and are nearly symmetric in the low-alpha range.

### Evidence for disrupted electrophysiologic brain activity in normal EEGs of DRFE patients

**Figure 4:**
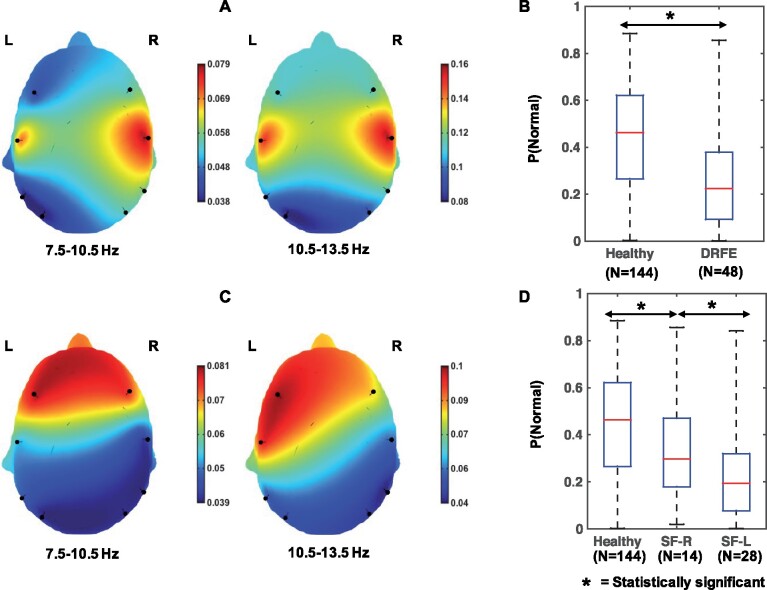
**Disrupted normal brain function in the DRFE population.** (**A**) Head plots illustrating the location-specific Wasserstein distances between the CDFs of log-transformed alpha power features of healthy and DRFE individuals. (**B**) Boxplots of window-level probability-of-normality estimates for healthy and DRFE individuals. (**C**) Head plots illustrating the location-specific Wasserstein distances between the CDFs of log-transformed alpha power features of right-handed DRFE individuals who had right-hemispheric seizures and those who had left-hemispheric seizures. (**D**) Boxplots of the window-level probability of normality estimates for healthy and right-handed DRFE individuals where the DRFE individuals are further stratified based on the hemisphere generating seizures. In (**B**) and (**D**), the numbers *N* indicate the number of participants whose data were used in generating the boxplot.

The primary goal of this study is to understand whether there are subtle abnormalities in the visually classified normal EEGs of DRFE patients. To study this, we analysed how the distributions of the log-transformed alpha-power fractions in the healthy and DRFE individuals differed using (i) their CDFs and (ii) the probability-of-normality values. Figure 4A illustrates the differences between the log-transformed alpha-power fractions of healthy and DRFE individuals, based on the Wasserstein distance between CDFs. We observed that the differences are notable in frontal and temporal regions. Particularly, the alpha power values were significantly lower in the DRFE population when compared with the healthy population, and this was highlighted in the frontal and temporal regions. We then used the probability-of-normality values described previously to characterize the differences across multiple brain regions. Figure 4B shows the boxplots of the window-level probability-of-normality values in the healthy and DRFE populations. We found that the probability-of-normality values are significantly lower in the DRFE population compared to the healthy population (*P* < 0.05 based on the Wilcoxon rank-sum test).

### The side of seizure focus impacts the extent of brain activity disruptions in focal epilepsy

To understand the contribution of the seizure focus to disruptions in brain activity, we analysed how the distributions of the log-transformed alpha-power fractions within the right-handed DRFE patients differed based on the hemisphere generating seizures. As described previously, the left side is generally the dominant hemisphere in right-handed DRFE patients. Figure 4C illustrates the spatial patterns of the differences between the log-transformed alpha-power fractions of right-handed DRFE individuals who had right-hemispheric seizures and those who had left-hemispheric seizures, based on the Wasserstein distance between CDFs. We found that the differences are emphasized notably in frontal and temporal regions. Similar to the differences observed between healthy and DRFE patients, the alpha power values were significantly lower in the individuals with left-hemispheric seizures compared to those with right-hemispheric seizures. We again utilized the probability-of-normality values to demonstrate this. Figure 4D shows the boxplots of the window-level probability-of-normality values between the two groups. We found that the probability-of-normality values were significantly lower in right-handed DRFE patients with left-hemispheric seizures compared to those with right-hemispheric seizures (*P* < 0.05, Wilcoxon rank-sum test).

### Visually reviewed normal EEGs can help diagnose DRFE and lateralize seizure focus

**Figure 5:**
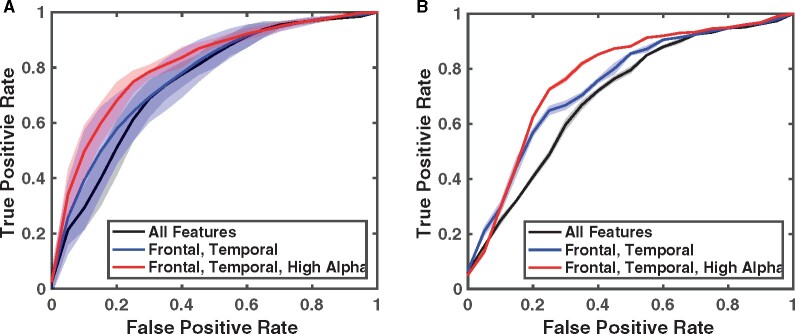
**Receiver operating characteristic (ROC) curves for the two classification tasks.** (**A**) ROC curves for classifying healthy individuals and DRFE patients. (**B**) ROC curves for classifying seizure generating side of the brain in right-handed DRFE patients. In both (**A**) and (**B**), red, blue and black curves indicate classifications using features extracted in three ways: (i) from all regions and both alpha bands; (ii) frontal–temporal regions and both alpha bands; and (iii) frontal–temporal regions and the high alpha band only, respectively. Furthermore, solid lines indicate average values obtained using the tenfold cross-validation, and shaded areas indicate 95% confidence intervals.

Next, to evaluate the potential clinical usefulness of alpha activity abnormalities and lateralize the hemisphere of the seizure focus, we performed two classification experiments: (i) classifying healthy and DRFE individuals, and (ii) classifying the seizure-generating side of the brain, both using the classification framework described previously. Figure 5A and B illustrates the ROC curves for the two classification tasks. Furthermore, Table 2 displays the goodness-of-fit metrics for the classification tasks, calculated based on the ROC analysis. We found that the probability-of-normality values derived based on a healthy sample can be used to differentiate previously unseen healthy and DRFE patients (AUC = 0.77). Similarly, we found that the same probability values can also be used to differentiate the seizure-generating side of the brain in a previously unseen DRFE population (AUC = 0.68). In both cases, the classification performance was significantly better than chance (AUC > 0.5) and showed minimal variation in a tenfold cross-validation scheme. We also found that the probability-of-normality values based on the frontal and temporal alpha features provided marginally better results in both the classification tasks compared to all regions (mean AUC improvements of 0.01 and 0.05, respectively). In addition, we observed additional improvements in the AUC when we further restricted the features to the high-alpha frequency band alone (mean AUC improvements of 0.05 and 0.04, respectively).

**Table 2:** Cross-validated goodness-of-fit metrics for classifying (i) healthy and DRFE individuals, and (ii) the seizure-generating side of the brain

Task	Regions	Frequency (Hz)	AUC	Precision	Recall	F1
Healthy vs. DRFE	All	7.5–13.5	0.77 (0.02)	71.42 (2.69)	73.96 (4.31)	72.57 (2.20)
Healthy vs. DRFE	Frontal, temporal	7.5–13.5	0.78 (0.04)	71.79 (5.39)	76.25 (10.50)	73.33 (4.06)
Healthy vs. DRFE	Frontal, temporal	10.5–13.5	0.83 (0.02)	79.41 (3.65)	73.54 (3.55)	76.24 (1.49)
Seizure focus	All	7.5–13.5	0.68 (0.00)	72.73 (0.00)	85.71 (0.00)	78.69 (0.00)
Seizure focus	Frontal, temporal	7.5–13.5	0.73 (0.01)	74.19 (2.81)	93.21 (6.83)	82.39 (1.45)
Seizure focus	Frontal, temporal	10.5–13.5	0.77 (0.00)	80.00 (0.00)	85.71 (0.00)	82.76 (0.00)

We list the goodness-of-fit metrics (AUC, precision, recall and F1-score) obtained for the test dataset, for the different evaluations using the alpha features: (i) from all regions and both alpha bands; (ii) frontal–temporal regions and both alpha bands; and (iii) frontal–temporal regions and the high alpha band only, respectively. Average values and standard deviations (within parentheses) were computed using tenfold cross-validation.

### Epilepsy associated disruptions in the alpha rhythm are significant when controlled for age, acquisition system differences and ASMs

Finally, we sought to distinguish the contribution of chronic DRE in the observed spectral changes of the alpha rhythm from the contributions of age and ASMs. In these evaluations, we calculated probability-of-normality estimates using the normalized alpha power features extracted from all brain regions and both low and high alpha frequency bands.

**Figure 6:**
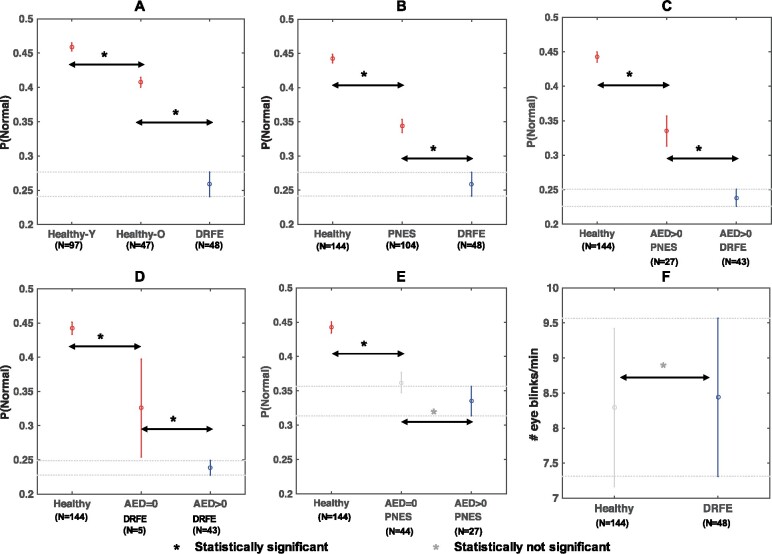
**Multiple comparisons analyzing the contributions of age, acquisition systems, and antiepileptic drugs (AEDs). Figures show the point estimates and comparison intervals of the mean probability of normality values.** (**A**) A comparison between healthy and DRFE individuals where the healthy individuals are stratified based on the age-group: Young (Healthy-Y): 20–35, and Old (Healthy-O): 59–77. (**B**) A comparison between healthy individuals and patients with PNES and DRFE, where the EEGs of both patient groups were acquired using the same acquisition system. (**C**) A comparison between healthy and patients with PNES and DRFE, where the patients were limited to those consuming AEDs. (**D**) A comparison between healthy and DRFE individuals where the DRFE individuals are stratified based on the number of AEDs consumed at the time of EEG. (**E**) A comparison between healthy and PNES individuals where the PNES individuals are stratified based on the number of AEDs consumed at the time of EEG. (**F**) A comparison between healthy and DRFE individuals based on the rate of eye blinks per minute. The numbers *N* indicate the number of participants whose data were used in the analyses.

*Effects of* *ageing* *versu* *s* *DRFE*: In this analysis, we used the alpha features of healthy-young individuals (age: 20–35, *N* = 97) for characterizing normal brain function based on CDFs. Then, using those CDFs, we computed the window-level probability-of-normality estimates (as described in Methods) for healthy-old individuals (age: 59–77, *N* = 47) and DRFE patients (*N* = 48). Figure 6A illustrates the results of multiple comparisons performed on those probability-of-normality values between the different groups. Note that we used the data of healthy-young individuals as a reference in this analysis. We found that the differences between young and old healthy individuals, and the differences between healthy and DRFE individuals, were both significant (*P* < 0.01). We also observed that the mean window-level probability-of-normality of DRFE patients was significantly lower than the means of the other two groups.

*Effects of acquisition system differences versu* *s* *DRFE*: In this analysis, we performed a three-way comparison between the healthy (*N* = 144), PNES (*N* = 104) and DRFE (*N* = 48) populations. Note that the EEGs of PNES and DRFE patients were recorded using the same acquisition system (XLTEK, Inc.). Figure 6B illustrates the results of multiple comparisons performed on those probability-of-normality values between the different groups. Consistent with our hypothesis, we found a difference between PNES and DRFE (*P* < 0.01). Interestingly, we also found a difference between PNES and normal controls (*P* < 0.01). We also observed that the mean window-level probability-of-normality of PNES patients was significantly lower than those of healthy individuals, and the mean probability-of-normality of DRFE patients was significantly lower than the means of both healthy and PNES groups.

We performed another comparison to further homogenize the PNES and DRFE populations. We selected subgroups who were taking ASMs within PNES (*N* = 27) and DRFE (*N* = 43) populations. Noteworthy is that these two populations share similar characteristics with respect to acquisition system, ASMs, and age range (PNES age range: 19–62, DRFE age range: 18–66). Figure 6C illustrates the results of multiple comparisons performed on those probability-of-normality values between the two groups. We found that the difference between PNES and DRFE is still significant (*P* < 0.01) when all confounders are controlled for.

*Effects of ASMs in DRFE*: In this analysis, we used the alpha features of all healthy individuals (*N* = 144) for characterizing normal brain function. Then, using those CDFs, we computed the window-level probability-of-normality values for two groups of DRFE patients: patients not taking any ASMs (*N* = 5) and patients taking ASMs (*N* = 43). Figure 6D illustrates the results of multiple comparisons performed on those probability-of-normality estimates between the different groups. Like all previous analyses, we used the data of healthy individuals as a reference in this analysis. We found that the difference between DRFE individuals based on whether or not they took ASMs was significant (*P* < 0.05). However, the mean probability-of-normality of DRFE individuals was still lower than that of the healthy individuals, regardless of whether or not they took ASMs. Furthermore, the variance of the probability-of-normality values of the DRFE patients not taking ASMs was notably large because of the small sample size (*N* = 5).

*Effects of ASMs in PNES*: We performed a similar comparison within the PNES population to address the sample size limitations. We identified 44 PNES patients who were not taking any ASMs and 27 taking ASMs and computed the window-level probability-of-normality values with respect to the healthy population. The results of multiple comparisons between those groups are illustrated in Fig. 6E. Although the effect of ASMs appears to be in the same direction (i.e. reduction in probability-of-normality), in contrast to the previous result, the difference with respect to consumption of ASMs was not significant within the PNES patients (*P* = 0.19).

*Comparison between major ASMs*: In this analysis, we divided the DRFE population based on the consumption of two ASMs: levetiracetam (*N* = 25) or lamotrigine (*N* = 19). We computed the window-level probability-of-normality values for those two groups of DRFE patients based on the CDFs obtained from the healthy population. Supplementary Fig. 1A illustrates the results of multiple comparisons performed on those probability-of-normality estimates between the different groups. We found that the difference between DRFE individuals based on whether they took levetiracetam or lamotrigine was not significant (*P* = 0.25).

*Comparison between arousal states*: We utilized the rate of eye blinks as a surrogate measure for drowsiness and compared the number of eye blinks within a minute between healthy and DRFE populations. Figure 6F shows a comparison between the rates of eye blinks of the two groups where the criterion that the upstrokes and downstrokes of the eye blinks were significantly correlated (*R*^2^ > 0.90) was used to identify robust eye blinks. We found that the two populations were not significantly different with respect to the rate of eye blinks (*P* = 0.89). A comparison between all potential eye blinks is provided in Supplementary Fig. 1B, where the difference is still not significant (*P* = 0.23).

## Discussion

### Main contributions of the study

In this study, we investigated whether visually classified normal EEGs of patients with epilepsy contain subtle abnormalities that may have diagnostic and clinical value. We conducted this retrospective study using the scalp EEG recordings of 48 patients with DRFE that were visually classified as normal EEG by expert review and the scalp EEGs of 144 age-matched healthy individuals. We extracted alpha power-related measures from eyes-closed (EC), awake segments in the EEGs of the healthy population and used them to represent normal brain activity. We then analysed how the same alpha power-related measures in the DRFE population differed from the healthy controls. Our analyses indicated that (i) alpha power is significantly reduced in DRFE compared to healthy controls and (ii) alpha power of right-handed DRFE patients with left hemispheric seizures is significantly lower compared to those with right hemispheric seizures. We also utilized these findings in a classification framework to classify (i) whether an EEG was recorded from an epilepsy patient and (ii) if so, the seizure generating side of the patient's brain by using EEG recordings that do not contain any epileptiform activity. A 10-fold cross-validation approach achieved mean AUC values of 0.83 and 0.77 for the respective classification tasks (when high-alpha features from frontal-temporal regions were used). These findings suggest that EEG measures representing normal brain function may be useful in the diagnosis of epilepsy even when the EEG is free of epileptiform activity. This finding is significant because the ability to diagnose epilepsy at the earliest possible time can prevent significant delays in treatment and can support more efficient triage of patients to costly in-hospital monitoring studies. In that context, our study presents a promising research direction in the treatment of epilepsy.

We note that several previous studies have reported alpha spectral abnormalities in epilepsy and suggested the potential diagnostic value in routine EEG studies.^22–24^ Building on those existing studies, our study makes several unique contributions: (i) evaluation of alpha-rhythm abnormalities in DRFE using multiple EEG windows extracted from routine EEG recordings that were visually classified as normal; (ii) characterizing the spatial patterns of alpha-spectral abnormalities in DRFE and their value in identifying the seizure generating hemisphere; (iii) development of an approach to combine alpha-spectral abnormalities from several channels and windows to generate the probability of normality measures; (iv) classification analysis to clearly evaluate the diagnostic value of alpha-spectral abnormalities; and (v) validating our findings against confounders such as acquisition system/condition differences, age and medications.

### Spatial and spectral patterns of the alpha abnormalities in DRFE

Our results showed significant abnormalities in the normalized spectral power of the alpha rhythm in the frontal and temporal regions of DRFE patients (Fig. 4A and C). This observation was further highlighted in the classification tasks; we found that using the alpha features extracted from frontal and temporal regions provided marginally better classification performances compared to using the same features extracted from all brain regions. These observations suggest that the alpha abnormalities in DRFE are of focal nature and display frontal spreading. We surmise that such characteristic changes in the alpha rhythm could have been modulated by the specific epilepsy syndrome because the majority of the DRFE patients (38 out of 48) had frontal or temporal seizure onset. Prior studies have also reported similar findings indicating the frontal spread of alpha-rhythm alterations in focal epilepsy and suggested that this effect could be commensurate with the extent of cortico-thalamic dysfunction.^22^

Our findings also agree with previously described slowing of the alpha rhythm observed in the presence of neurological diseases, including epilepsy.^22^ Headplots showed in Fig. 4A and C indicate that the differences between healthy and DRFE populations and the differences due to the side of seizure focus in the DRFE population are both greater in the high alpha band (10.5–13.5 Hz) compared to the low alpha band (7.5–10.5 Hz), based on the Wasserstein distance between CDFs. This finding was also signified in the classification performances; we found that using the spectral power features extracted from the high alpha band provided better classification performances in both the classification tasks compared to using the entire alpha band.

### Analysis of confounders

EEG spectral abnormalities related to neurological diseases are typically confounded by age-related changes and changes induced by certain medications.^20^ Furthermore, the EEGs of the two main populations in this study, healthy control and DRFE, were acquired using different systems under different conditions. All of the above variables are potential confounders in our study, and we conducted several experiments to understand the contributions of those confounders. First, we showed that the alpha abnormalities we observed in the normal EEGs of DRFE patients were significant when compared with changes related to ageing, using a multiple comparison approach (Fig. 6A). Second, we analysed the contribution of acquisition system/environment differences using two experiments: (i) we compared the probability-of-normality measures estimated for DRFE patients and PNES patients whose EEGs were recorded using the same system under similar conditions as the DRFE patients (Fig. 6B), and (ii) a similar comparison between the same patient groups but limited only to those receiving ASMs (Fig. 6C). Our results indicated that the differences between PNES and DRFE patients were significant in both the experiments, although surprisingly, there were sizeable differences between healthy individuals and PNES patients. We believe that the differences between healthy individuals and PNES patients are attributable to acquisition system differences, medications, psychiatric comorbidities of PNES (Supplementary Table 2), and possibly the PNES aetiology itself.

Noteworthy is that all variables including, acquisition system/environment, age and ASMs, were similar in the second experiment between PNES and DRFE patients (Fig. 6C). A significant difference between those two groups strongly supports our main hypothesis that there are significant disruptions in the alpha-power-related characteristics of DRFE patients. To further support this result, we performed a classification experiment between PNES and DRFE patients, both taking ASMs, using the same framework described in Fig. 2D. The results, illustrated in Supplementary Fig. 2 and Table 3, indicate an AUC of 0.62 and F1-scores above 70% in classifying those two groups. Based on these findings, we contend that the difference between healthy and DRFE is unlikely to be less significant than the aforementioned results, although confirmation will require EEGs of healthy individuals recorded using the same acquisition system. We then analysed the effects of ASMs in DRFE and PNES populations separately (Fig. 6D and E). Although our results are not consistent, the comparison within the PNES population (Fig. 6D), supported by substantial sample size, suggests that the ASM-induced changes in the EEG spectral power of the alpha rhythm are not significant enough to disprove our main hypothesis. However, we note that the same analysis in the DRFE population can benefit from additional samples to increase the statistical power. Finally, we analysed the differences in arousal states between the healthy and DRFE populations to clarify whether DRFE patients are in a more drowsy state during EEG recording because of medications, previous seizures, and sleep deprivation. Specifically, we compared the rate of eyeblinks as a measure of drowsiness.^35^^,^^36^ Our results (Fig. 6F) showed that the difference is not significant enough to be of concern. These findings, taken together, suggest that the EEGs of DRFE patients, which were determined to be normal based on visual review, in fact, contain strong pathological correlates that may be sufficient to support clinical use.

### Methodological contributions

We developed a probabilistic approach to characterize normal brain function using EEG features based on the alpha rhythm extracted from eight bipolar channels. This involves computing the CDFs of log-transformed alpha power features in the individual channels of 10-s EEG windows and using those CDFs to assign a window-level probability-of-normality estimate to new windows. We combined the individual channel-based values to obtain a combined probability-of-normality estimate for each window. Furthermore, we applied a maximum likelihood approach to aggregate the window-level probability-of-normality values of a participant's entire EEG recording to obtain a probability-of-normality estimate for the participant. These values were then used for classification purposes. Our approach can be considered as an anomaly detection technique wherein we estimate whether a new sample is anomalous compared to the reference population. An advantage of this approach is that it can be developed using the data of the reference population alone without requiring data from anomalous samples. Furthermore, our approach presents a general paradigm for EEG-based anomaly detection, which can be beneficial for other EEG applications such as seizure forecasting.^45^ In addition, our approach to characterize the health of brain function using the alpha rhythm can form the basis for the growing area of research on EEG and brain health^46^ and can be used to study a variety of other neurological conditions.

### Study limitations and future work

Our participants were limited to healthy individuals and DRFE and PNES patients. However, a normal EEG could be recorded from a patient with any number of other neurologic or psychiatric diseases. As such, although our study establishes the feasibility of using alpha spectral features in aiding the clinical diagnosis of epilepsy,^47^ a population-level study including EEGs from a heterogeneous sample is necessary to accurately evaluate the clinical utility of our findings. In addition, our analysis relied on expert EEG annotations regarding EC and EO conditions, awake and sleep, and bad channels. Such annotations are time-consuming, costly, susceptible to human error, and clearly not scalable. Fully automated approaches that can analyse raw EEG data without requiring expert annotations of specific events can enable large-scale studies, eliminate reviewer biases, and identify novel EEG features and advance scientific knowledge. Such automated approaches may also augment the visual review of epileptologists by providing focussed inputs and help reduce physician burnout.^48^

Another limitation of our study is that the EEGs of two populations, healthy and DRFE, were acquired using different systems under different conditions. To address this limitation as best we could, (i) we undertook the same preprocessing steps for both the EEG datasets, and (ii) we used the fraction of alpha power within the wideband of 0.5–40 Hz to mask any subject-specific differences in total signal power. In addition, we also demonstrated using a population of PNES patients that the differences between healthy and DRFE populations are not entirely due to acquisition system/environment differences. However, EEGs of both controls and patients recorded using the same acquisition system are necessary to confirm our results without this confounder. Our future efforts will investigate this possibility.

Finally, there is substantial evidence in the literature suggesting the value of EEG functional connectivity measures in increasing the diagnostic value of normal EEG segments.^15–18^ An investigation focussing on whether functional connectivity measures and the alpha rhythm can complement each other and improve the combined diagnostic value is worth exploring. The combination of these two measures can increase the specificity and enable a broader set of possibilities including, (i) differentiation of focal versus generalized epilepsy; (ii) differentiation of drug resistance versus drug-responsive epilepsy; and (iii) differentiation of nonepileptic seizures from healthy and epilepsy, and allow clinicians to individualize treatments based on disease subtype, particularly in the absence of epileptiform abnormalities in routine or prolonged EEG studies.

### Envisioned clinical applications

Upon further validation using population-level datasets and sufficient automation of the proposed approach, we envision that a clinical decision support tool will be developed and deployed for prospective evaluation. This tool will provide probability-of-normality estimates at a patient level to guide decisions regarding costly inpatient EEG studies in the absence of known epileptiform abnormalities. This could prove particularly useful in obtaining payer authorizations for prolonged EEG studies in the case of normal routine EEGs. Furthermore, this tool can also help in the lateralization of focal epilepsy and can potentially impact presurgical evaluations. Although there is a possibility of misclassification, the risk associated with such errors is minimal because prolonged EEG studies can only improve the diagnostic yield. As such, we believe that the proposed approach and the findings have the potential to directly impact clinical practice and therefore warrant further investigation.

## Conclusion

EEG-based diagnosis of epilepsy, which is the gold-standard approach, relies on visual identification of epileptiform activity. However, epileptiform activity may not be recorded in a short EEG recording session, and that can cause delays in the delivery of clinical care. Unfortunately, such scenarios are common in the clinic; approximately 50% of the EEGs recorded from patients with seizures are deemed normal based on expert visual review. In this study, we investigated the possibilities of diagnosing DRFE and lateralizing seizure focus based on normal EEGs using a semi-automated approach. Our results support the hypothesis that EEG-based measures of normal brain function, based on the alpha rhythm, can help diagnose DRFE and lateralize seizure focus when an EEG does not contain any epileptiform activity, recorded seizures, or other non-specific abnormalities. Based on these findings, we further hypothesize that such findings in a normal EEG can suggest a higher pre-test probability for epilepsy when an individual is screened for epilepsy for the first time. In addition, our findings also suggest that automated analyses of scalp EEG can help in developing scalable and cost-effective approaches for advancing the current state of clinical electrophysiology. However, prospective studies and addressing the identified limitations of our work are necessary to fully understand the clinical value of these hypotheses. Going forward, our efforts will focus on expanding the study to population-level datasets, including other biomarkers of normal brain function, developing fully automated methods, and addressing systemic biases introduced by EEG acquisition systems.

## Supplementary material

Supplementary material is available at *Brain Communications* online.

## Supplementary Material

fcab102_Supplementary_Data

## Abbreviations

ASM =: anti-seizure medications
AUC =: area under the curve
CDF =: cumulative density function
DRE =: drug-resistant epilepsy
DRFE =: drug-resistant focal epilepsy
EC =: eyes closed
EO =: eyes open
PNES =: psychogenic nonepileptic seizures
ROC =: receiver operating characteristics
