# Supplementary material for: Characterizing the electrophysiological abnormalities in visually reviewed normal EEGs of drug-resistant focal epilepsy patients
Source: Brain Commun. 2021 May 14;3(2):fcab102. doi: 10.1093/braincomms/fcab102 (PMC8196245; doi:10.1093/braincomms/fcab102)
Supplement: fcab102_Supplementary_Data [file fcab102_supplementary_data.zip › Original Submission.pdf]

OXFORD UNIVERSITY PRESS | Brain Communications

Characterizing the Electrophysiological Abnormalities in Visually-reviewed Normal EEGs of Drug-Resistant Focal Epilepsy Patients

Journal:	*Brain Communications*
Manuscript ID	BRAINCOM-2021-054
Manuscript Type:	Original Article
Date Submitted by the Author:	07-Feb-2021
Complete List of Authors:	Varatharajah, Yogatheesan; University of Illinois at Urbana-Champaign, The Department of Bioengineering<br>Berry, Brent; Mayo Clinic, Neurology; Mayo Clinic Minnesota, Biomedical Engineering and Physiology<br>Boney, Joseph; Mayo Clinic<br>Balzekas, Irena; Mayo Clinic<br>Pal Attia, Tal; Mayo Clinic<br>Kremen, Vaclav; Mayo Clinic, Neurology; Mayo Clinic Minnesota, Biomedical Engineering and Physiology<br>Brinkmann, Benjamin; Mayo Clinic, Neurology<br>Iyer, Ravishankar; University of Illinois at Urbana-Champaign<br>Worrell, Gregory; Mayo Clinic, Neurology and Division of Epilepsy
Keywords:	Drug-resistant Epilepsy, Alpha rhythm, Normal EEG, Brain health

SCHOLARONE™
Manuscripts

Characterizing the Electrophysiological Abnormalities in Visually-reviewed Normal EEGs of Drug Resistant Focal Epilepsy Patients

Yogatheesan Varatharajah[1,2,4], Brent Berry[2], Boney Joseph[2], Irena Balzekas[2], Tal Pal Attia[2], Vaclav Kremen[2,3], Benjamin Brinkmann[2], Ravishankar Iyer[4], & Gregory Worrell[2]
[1] Department of Bioengineering, University of Illinois, Urbana, IL 61801, USA.
[2] Mayo Systems Electrophysiology Laboratory, Department of Neurology, Mayo Clinic, Rochester MN, 55905, USA.
[3] Czech Institute of Informatics, Robotics and Cybernetics, Czech Technical University in Prague, Prague, Czech Republic.
[4] Electrical and Computer Engineering, University of Illinois, Urbana, IL 61801, USA.

Abstract

Introduction: Routine scalp EEG is important in the clinical diagnosis and management of epilepsy. However, a normal scalp EEG (based on expert visual review) recorded from a patient with epilepsy can cause delays in clinical care delivery. Here we hypothesized that even normal EEGs might contain subtle electrophysiological clues of epilepsy. Specifically, we investigated a) whether there are indicators of disrupted brain functions in normal EEGs of epilepsy patients compared to healthy controls, and b) whether such disruptions are modulated by the side of brain generating seizures in focal epilepsy.

Methods: We analyzed awake scalp EEG recordings of age matched groups of 144 healthy individuals and 48 individuals with drug-resistant focal epilepsy (DRFE) who had normal scalp EEGs. After preprocessing, using a bipolar montage of eight channels, we extracted the fraction of spectral power in alpha band (8-13 Hz) relative to a wide band of 0.5-40 Hz, within 10-second windows. We then analyzed the extracted features for a) the extent to which DRFE patients differed from healthy patients, and b) whether differences within the DRFE patients were related to the hemisphere generating seizures. We used those differences to classify whether an EEG is likely to have been recorded from a DRFE patient, and if so, the epileptogenic hemisphere. Furthermore, we tested the significance of these differences while controlling for confounders such as age and medications.

Results: We found that the fraction of alpha power is generally reduced a) in DRFE compared to healthy controls, and b) in right-handed DRFE patients with left hemispheric seizures compared to those with right hemispheric seizures, and that the differences are most prominent in the frontal and temporal lobes. The fraction of alpha power yielded an AUC of 0.83 in distinguishing DRFE patients from healthy individuals, and an AUC of 0.77 in identifying the epileptic hemisphere in DRFE patients. Furthermore, the observed differences in the fraction of alpha power between healthy and DRFE are greater when compared with alterations in the alpha rhythm attributable to age and drug therapy.

Conclusion: Our results support that EEG-based measures of normal brain function, such as the normalized spectral power of alpha activity, may help identify patients with epilepsy even when an EEG does not contain any epileptiform activity, recorded seizures, or other abnormalities. Although alpha rhythm abnormalities are unlikely to be disease specific, we propose that such

For Review Only

abnormalities may provide a higher pre-test probability for epilepsy when an individual is screened for epilepsy for the first time and has a normal EEG on visual assessment.

For Review Only

Introduction

Epilepsy is a neurological disease characterized by unprovoked seizures and affects 1% of the global population [1]. Epileptologists assess the potential for epilepsy and related conditions by visually identifying abnormal activity (also known as epileptiform activity) in a short scalp electroencephalography (EEG) recording session (~20–60 minutes). A positive screen, the presence of abnormal epileptiform EEG transients, is typically followed by the initiation of anti-seizure medication and further evaluation. However, this initial assessment is not always sensitive enough, as epileptiform activity may not be recorded in a short EEG session. Unfortunately, such scenarios are very common in clinical setting [2], and some patients with drug-resistant epilepsy (DRE) have normal EEGs on expert visual review (i.e., the EEGs did not contain any epileptiform activity) [3, 4]. The inability to find evidence for epilepsy at the earliest possible time can cause delay in delivering appropriate and early clinical care [5]. Even after anti-seizure medications are initiated approximately 1/3 of people will not completely respond and continue to have seizures despite multiple different medication trials. Each medication trial can take months, and thus puts the patient at continued risk for seizure related injuries and comorbidities [6]. Furthermore, and in patients determined to have DRE, a more comprehensive evaluation is indicated to determine if they are candidates for non-pharmacological therapies, e.g. surgery and electrical stimulation. Thus, more rapid diagnosis of epilepsy, and in particular DRE, is needed. In this study, we hypothesize that even short EEG recordings might contain subtle electrophysiological abnormalities that can indicate the possibility of epilepsy even when recognizable epileptiform activity is absent. If confirmed, this could improve the diagnostic yield of routine EEG and facilitate more sensitive, objective, and earlier diagnosis and treatment of epilepsy.

EEG is the primary method to diagnose epilepsy [7]. Electrodes are attached to an individual's scalp to record the brain electrical activity. In people with epilepsy, it is common to see transient voltage disruptions to the normal pattern of brain waves, even in the interictal recording when a patient is not having a seizure [8]. The most common abnormalities in brain activity associated with epilepsy are interictal spikes and sharp waves (IIS) [9]. IIS represent the summated excitatory and inhibitory postsynaptic potentials of a large population of neurons [10] and have similar underlying physiological causes. The difference in their appearances reflects the rapidity of neuronal synchronization and the way in which the epileptiform discharge spreads over the cortex. In addition to providing evidence for epilepsy, the spatial distribution of these events can identify epilepsy as having generalized or focal origins. In focal epilepsy, the distribution of IIS can help spatially map epileptogenic brain regions [11]. However, these abnormalities may not be observed in short EEG recordings for multiple reasons, e.g. they may be very infrequent and not captured on routine ~30 minute recording, they may originate from deeper brain structures like cingulate, hippocampus etc., they are activated only during sleep that was not recorded, or they involve an insufficient amount of cortex to be measurable on the scalp [12].

Pathologic changes such as neuronal loss and gliosis are common in chronic epilepsy, though the same neuronal-glia circuits underlying seizure generation may subserve normal brain functions [13, 14]. The cellular changes associated with epilepsy may be expected to cause subtle declines in EEG-based measures of normal brain function. The alpha rhythm observed on EEG during eyes-closed wakefulness is considered as a potential biomarker of normal brain function in adults and its frequency and power decrease with age [15]. It is theorized to arise through cortico-thalamic

For Review Only

interactions, and to reflect processes that subserve a vast range of cognitive processes, including attention and memory [16]. Alterations in the alpha rhythm have been observed in many neurological diseases including epilepsy, where it typically slows down and loses its characteristic anterior-to-posterior gradient proportionally with clinical severity [17]. Although alpha-rhythm-related abnormalities are well known in epilepsy, they are not included in diagnostic criteria because they lack specificity to any neurological disease [18, 19]. In addition, the analysis of alpha rhythm in the EEGs of epilepsy patients is further complicated by its changes related to aging [15] and antiepileptic, antidepressant, and antipsychotic medications [20-22]. However, we propose that alpha-rhythm related abnormalities can provide a higher pre-test probability for epilepsy when an individual is screened for epilepsy. Alpha-rhythm abnormalities could therefore warrant additional testing when a patient's EEG does not contain epileptiform activity, e.g. prolonged EEG recording that includes sleep. Additionally, there is evidence that people with epilepsy who have seizures originating from their dominant hemisphere can experience relatively more disruptions in their normal brain function, compared to those with seizure foci in their non-dominant hemispheres [23]. Therefore, we further hypothesize that in addition to indicating the potential for epilepsy, alpha rhythm abnormalities can also help lateralize the seizure focus in focal epilepsy. In summary, our study investigates whether we can extract subtle clues of epilepsy in EEGs visually classified as normal.

To test our hypotheses, we analyzed the scalp EEG recordings of healthy individuals age matched to patients with DRFE who went through clinical evaluations at the Mayo Clinic. The EEGs of DRFE individuals were classificed as normal based on expert visual review performed by board-certified epileptologists. We preprocessed the EEGs to remove artifacts and performed spectral analysis to extract EEG features representing the fraction of spectral powers contained within the alpha band across the four major brain regions. We then used the extracted features to analyze, a) the extent to which DRFE patients deviated from healthy, and b) whether there are differences within the DRFE patients based on the hemisphere generating seizures. Furthermore, we analyzed whether these differences are significant when compared with confounders such as the individual's age, acquisition system differences, and antiepileptic drugs.

For Review Only

Materials and Methods

Our primary analyses utilized the scalp EEG data of 144 healthy individuals age matched to 48 patients with DRFE. We also utilized the normal scalp EEG data of 104 patients with psychogenic nonepileptic seizures (PNES) to analyze the effect of different EEG acquisition systems on our findings. We obtained the data on healthy individuals from the publicly available LEMON dataset [24] and the data of DRFE and PNES patients from clinical records at the Mayo Clinic, Rochester, Minnesota, USA.

Data from healthy individuals: The LEMON dataset consisted of scalp EEG recordings from 203 healthy individuals (median age 39, age range 20–77, 82 females) [24]. The participants were stratified between *young* and *old* groups with age-ranges 20–35 and 59–77, respectively. The EEGs were recorded using the BrainAmp MR plus recording system with the ActiCAP electrodes (both from Brain Products GmbH, Gilching, Germany) including 62 channels according to the standard 10-10 localization system [25]. The data were originally recorded at a sampling rate of 2500 Hz and then downsampled to 250 Hz, and each EEG session comprised 8 eyes-closed (EC) and 8 eyes-open (EO) segments, each 60 seconds long. We rejected the channels that were determined as outlier channels by the investigators of the LEMON study [24]. We excluded the data of 59 individuals from our study because some channels required for our analyses were either not available or deemed outlier channels.

Data from DRFE and PNES patients: We obtained scalp-EEG recordings from 48 individuals with DRFE (median age 39, age range 18–66, 25 females) and 104 individuals with PNES (median age 30.5, age range 18–62, 60 females) that were performed as part of their clinical evaluations. Our study was approved by the Mayo Clinic Institutional Review Board and patients provided informed consent. The EEGs were recorded using the XLTEK EMU40EX headbox (from Natus Medical Incorporated, Oakville, Ontario, Canada) with the WaveGuard Original EEG cap (from Ant Neuro GmbH, Berlin, Germany) including 31 channels according to the extended 10–20 localization system [26] at a sampling rate of 256 Hz. The EEGs were visually reviewed by board-certified epileptologists and classified as normal. We selected EC segments based on the annotations made by EEG technologists during clinical video-EEG review.

Stratifying participants for further analyses: Our first analysis focused on differences between epilepsy patients and healthy individuals. We utilized the data of all 144 healthy individuals from the LEMON study and 48 Mayo patients for this analysis. Our second analysis focused on differences based on the seizure-generating hemisphere of the brain in epilepsy patients. In addition to the EEG features, we utilized handedness (right or left) to determine the dominant hemisphere. Of the 48 epilepsy patients, 43 were right-handed and 5 were left-handed. Furthermore, although there is evidence that dominant hemisphere of right-handed individuals is generally deterministic (i.e., the left side), the dominant hemisphere of left-handed individuals is nondeterministic (can be the left or the right side) [27]. Therefore, we excluded the a) left-handed patients, because of insufficient sample size and the nondeterministic nature of their dominant hemisphere, and b) one right-handed patient who had a central midline (non-lateralized) seizure onset, from this analysis. As a result, the second analysis utilized the EEG data of 42 right-handed DRFE patients (28 patients with seizure focus on the left side, and 14 patients with seizure focus on the right side). Third, we compared the effect of age against the group differences between

For Review Only

healthy and DRFE individuals. We divided the healthy population into young (97 individuals between ages 20 and 35) and old (47 individuals between ages 59 and 77) populations and compared them with the DRFE patients. Fourth, to analyze whether the group differences between healthy and DRFE individuals were distinct from acquisition system differences, we performed a 3-way comparison between all healthy, PNES, and DRFE individuals. Here, the EEGs of PNES and DRFE patients were recorded using the same acquisition system. Finally, we compared the effects of antiepileptic drugs (AEDs) in two ways: a) differences between DRFE patients who were not taking any AEDs (5 patients) compared to those who did (43 patients) and b) differences between DRFE patients who were taking either levetiracetam (25 patients) or lamotrigine (19 patients). See supplementary Table 1 for more details.

EEG preprocessing: Further preprocessing was done in EEGLAB for MATLAB [28]. First, the EEGs were bandpass-filtered within 1–45 Hz (8th order, Butterworth filter). Next, an independent component analysis was performed, and components reflecting eye movement, eye blink, or heartbeat-related artifacts were removed, and bad channels were rejected, all according to the widely-recognized Makoto's EEG preprocessing pipeline [29]. The retained independent components were back-projected to sensor space for further analysis.

Selection of EEG channels: Because the EEG data of healthy individuals were recorded using a 62-channel 10-10 system, we selected a subset of channels that matched the 10-20 system used to record the EEG data from epilepsy patients. Within the EEG data of selected channels from the healthy and patient populations, we selected 4 bipolar pairs of electrodes from each hemisphere, producing 8 channels of EEG data for each participant. Table 1 shows the electrodes that were used to form the bipolar montage representing each major brain region and hemisphere.

Table 1: Channels used to form the bipolar montage representing each major brain region and hemisphere.

Left Hemisphere	Right Hemisphere
F7-F3	F8-F4
T7-C3	T8-C4
P7-P3	P8-P4
O1-P3	O2-P4

Extracting spectral features: We first normalized (z-scored) each channel separately within each segment and divided each segment into 10-second non-overlapping windows (Figure 1A, only 5s long EEGs are showed for visual clarity). Note that the number of 10-second windows was different for each participant in the DRFE and PNES populations. We then computed the power spectrum for each window using the multitaper spectral estimation methods implemented in the Chronux toolbox [30]. In order to eliminate the subject-specific differences in total signal power, we also normalized all the calculated power values by using the total power of the signal within 0.5–40 Hz (Figure 1B). We then separately aggregated the normalized power within two frequency bands: low-alpha (7.5–10.5 Hz) and high-alpha (10.5–13.5 Hz); such a division of the alpha band allows the detection of slowing (more power in low-alpha than in high-alpha) as well as the overall reduction in alpha power. As a result, each calculated feature was a fraction of the total power

For Review Only

within one of the alpha bands and each 10-second window produced 16 features (8 channels x 2 frequency bands). Furthermore, each participant's EEG data produced $N_{EC} \times 16$ features, where N_{EC} is the number of EC windows. The healthy data consisted of an average of 47 EC windows (min=42, max=48), the DRFE patient data consisted of an average of 13 EC windows (min=4, max=69), and the PNES patient data consisted of an average of 11 EC windows (min=5, max=22).

Characterizing normal brain activity: We performed a log transformation of the features and used the cumulative density function (CDF) of the log-transformed features in the healthy population to characterize normal brain function. An example of this is pictorially illustrated in Figure 2A-C. Suppose that the CDF of a single log-transformed feature x_k ($k \in \{1,...,16\}$ is the feature number) in the healthy population (considering all participants) is denoted by $F(x_k)$, where $x_k \in [-\infty,0]$ and $F(x_k) \in [0,1]$. We hypothesize that the samples representing abnormal brain function will fall near the lower limit of x_k, suggesting that the likelihood of those samples coming from a population with normal brain function, which we refer to as the ***probability-of-normality***, is low. Therefore, the probability-of-normality for such samples will be close to zero, while the probability-of-normality for samples that are near the upper limit of x_k will be close to one. With this intuition, we use the CDF of x_k to estimate the probability-of-normality for a new sample x'_k, i.e., $P(x'_k \in Normal) = F(x'_k)$, as the CDF satisfies the aforementioned requirements. However, note that this estimation of probability-of-normality is based on a single feature x_k.

Calculating probability-of-normality for an EC window: To combine the values of all 16 features in an EC window, we assume that the individual estimations can be independently combined. By applying the independence assumption, we now derive a combined probability-of-normality as shown below.

$$P(x' \in Normal) = \sqrt[16]{\prod_{k=1}^{16} P(x'_k \in Normal)} = e^{\frac{\sum_{k=1}^{16} \log\left(P\left(x'_k \in Normal\right)\right)}{16}} \quad (1)$$

Note that we use the geometric mean of the product of the individual feature-based estimates as the combined estimate because the multiplication of 16 fractional numbers will produce a very small probability value. Furthremore, we calculated $P(x' \in Normal)$ described in (1) in the log domain to avoid numerical instability. This derivation provides a single probability value $P(x' \in Normal)$ for each EC window. Using the same approach, we can calculate window-level probability-of-normality values for all EC windows of all participants.

Individual-level probability estimation: To estimate whether a participant's EEG is *normal*, we used a maximum likelihood estimation based on the window-level probability-of-normality values. We model the window-level estimates of a participant P_i as independent observations made from a Bernoulli trial with an unknown probability π_i, where π_i is the probability-of-normality for participant P_i, i.e., $\pi_i = P(P_i \in Normal)$. Suppose that we use $x'(i,n)$ to denote the nth window of participant i, and $Y(i,n)$ to denote $P(x'(i,n) \in Normal)$. Then, an estimate of π_i that maximizes the likelihood function $\prod_{n=1}^{N(i)} \pi_i^{Y(i,n)} (1-\pi_i)^{(1-Y(i,n))}$ is given as the following.

$$\hat{\pi}_i = \frac{1}{N(i)} \sum_{n=1}^{N(i)} Y(i,n) \quad (2)$$

Computing differences between CDFs: In order to characterize the group differences based on the CDFs, we utilized the Wasserstein distance (or earth mover's distance) metric [31].

Visualizing group differences using boxplots: We performed the following operations to generate boxplots. First, we computed the CDFs of the 16 features (i.e., the log-transformed alpha-power fractions of an EC window) using the data of all 144 healthy individuals. Then, we calculated the window-level probability-of-normality estimates for the groups of individuals we were interested in comparing, using the computed CDFs (see previous sections for more information). Those values were then divided among the comparison groups to generate the boxplots. For reference, we also plot the probability-of-normality estimates for the same healthy individuals whose CDFs are used to generate those estimates.

Statistical tests: In order to test the statistical significance of the differences between two distributions, we used the Wilcoxon Rank Sum test [32]. Furthermore, we performed multiple comparisons between different stratifications in the study population using the Tukey's honestly significant difference procedure [33].

Headplots: Headplots illustrating the spatial distributions of features of interest were plotted using the headplot() function in the EEGLAB toolbox [28]. The headplots are generated using a spherically-splined field map of the feature of interest on a semi-realistic head model.

Classification framework: Figure 2D illustrates the approach we utilized for classifying healthy and DRFE patients using a ten-fold crossvalidation. During each crossvalidation, we selected a random sample of 96 healthy participants to generate the feature-specific CDFs characterizing normal brain function (i.e, training set). We used the data of the rest of the participants (48 healthy and 48 DRFE) to evaluate the classification potential of our approach (i.e., testing set). This scheme ensured that the training and testing datasets consisted of two disjoint sets of participants and that the testing set is class-balanced. First we computed the CDFs of the 16 features representing alpha power fractions in the EC windows using the healthy sample in the training set. Then, we computed the window-level probability-of-normality values for each EC window in the testing set. Then, we aggregated the window-level values to obtain individual-level values as described previously. By comparing individual-level probabilities with ground truth, we computed goodness-of-fit metrics for the classification task, separately for classifying a) healthy vs epilepsy and b) hemisphere with seizure focus.

Performance evaluation: We first plotted receiver operating characteristic (ROC) curves and calculated the area under ROC curve (AUC) to compare model performances. An optimal threshold on the ROC cruve was selected using the convex hull method [34]. That threshold was used to calculate precision, recall, and F1-score. The classification procedure was repeated ten times to calculate the mean and standard deviations of the metrics. In addition, because frontal and temporal lobe epilepsy are the most common forms of focal epilepsy, we performed the classification approach using the features from those two regions alone and compared the results with those obtained from the approach using features extracted from all regions.

For Review Only

Data availability: The data of healthy controls is already publicly available. The deidentified spectral features extracted from the EEGs of DRFE and PNES patients, and the software used to perform statistical analyses will be publicly shared upon acceptance of this manuscript.

For Review Only

Results

Characterizing normal brain function in the healthy population

We used the frequency domain features based on the alpha rhythm power to characterize normal brain function. Figure 3A illustrates the CDFs of log-transformed alpha-power fractions in the healthy population in EC and EO windows. The figure also highlights the differences between the four major brain regions with respect to the same features, where the CDF of each brain region was generated by taking the average of the CDFs of the respective regions in the left and right hemispheres. Our observations agree with the commonly-known characteristics of the alpha rhythm, i.e., a) it is posteriorly dominant, and b) its presence is amplified when the eyes are closed [18]. In addition, Figures 3B and 3C show the spatial patterns of the differences between EC and EO conditions in the two alpha bands, based on Wasserstein distances between the CDFs. We find that the differences are stronger in the posterior regions and are nearly symmetric.

Evidence for disrupted electrophysiologic brain activity in normal EEGs of DRFE patients

The primary goal of this study is to understand whether there are subtle abnormalities in the visually classified normal EEGs of DRFE patients. To study this, we analyzed how the distributions of the log-transformed alpha-power fractions in the healthy and DRFE individuals differed using a) their CDFs, and b) the probability-of-normality values. Figure 4A illustrates the differences between the log-transformed alpha-power fractions of healthy and DRFE individuals, based on the Wasserstein distance between CDFs. We observed that the differences are notable in frontal and temporal regions. Particularly, the alpha power values were significantly lower in the DRFE population when compared with the healthy population, and this was highlighted in the frontal and temporal regions. We then used the probability-of-normality values described previously to characterize the differences across multiple brain regions. Figure 4B shows the boxplots of the window-level probability-of-normality values in the healthy and DRFE populations. We found that the probability-of-normality values are significantly lower in the DRFE population compared to the healthy population ($p<0.05$ based on the Wilcoxon ranksum test).

The side of seizure focus impacts the extent of brain activity disruptions in focal epilepsy

To understand the contribution of the seizure focus to disruptions in brain activity, we analyzed how the distributions of the log-transformed alpha-power fractions within the right-handed DRFE patients differed based on the hemisphere generating seizures. As described previously, the left side is generally the dominant hemisphere in right-handed DRFE patients. Figure 4C illustrates the spatial patterns of the differences between the log-transformed alpha-power fractions of right-handed DRFE individuals who had right-hemispheric seizures and those who had left-hemispheric seizures, based on the Wasserstein distance between CDFs. We found that the differences are emphasized notably in frontal and temporal regions. Similar to the differences observed between healthy and DRFE patients, the alpha power values were significantly lower in the individuals with left-hemispheric seizures compared to those with right-hemispheric seizures. We again utilized the probability-of-normality values to demonstrate this. Figure 4D shows the boxplots of the window-level probability-of-normality values between the two groups. We found that the probability-of-

For Review Only

normality values were significantly lower in right-handed DRFE patients with left-hemispheric seizures compared to those with right-hemispheric seizures ($p<0.05$, Wilcoxon rank sum test).

Visually reviewed normal EEGs can help diagnose drug resistant focal epilepsy and lateralize seizure focus

Next, to evaluate the potential clinical usefulness of alpha activity abnormalities and lateralize the hemisphere of the seizure focus we performed two classification experiments: a) classifying healthy and DRFE individuals, b) classifying the seizure-generating side of the brain, both using the classification framework described previously. Figure 5A and 5B illustrate the ROC curves for the two classification tasks. Furthermore, Table 2 displays the goodness of fit metrics for the classification tasks, calculated based on the ROC analysis. We found that the probability-of-normality values derived based on a healthy sample can be used to differentiate previously unseen healthy and DRFE patients (AUC=0.77). Similarly, we found that the same probability values can also be used to differentiate the seizure-generating side of the brain in a previously unseen DRFE population (AUC=0.68). In both cases, the classification performance was significantly better than chance (AUC > 0.5) and showed minimal variation in a tenfold cross-validation scheme. We also found that the probability-of-normality values based on the frontal and temporal alpha features provided marginally better results in both the classification tasks compared to all regions (mean AUC improvements of 0.01 and 0.05, respectively). In addition, we observed additional improvments in the AUC when we further restricted the features to the high-alpha frequency band alone (mean AUC improvements of 0.05 and 0.04, respectively).

Table 2: Cross-validated goodness-of-fit metrics for classifying a) healthy and DRFE individuals and b) the seizure-generating side of the brain. We list the goodness-of-fit metrics (AUC, precision, recall, and F1-score) obtained for the test dataset, for the different evaluations using the alpha features: 1) from all regions and both alpha bands, 2) frontal- temporal regions and both alpha bands, and 3) frontal-temporal regions and high alpha band only, respectively. Average values and standard deviations (within parentheses) were computed using a tenfold cross validation.

Task	Regions	Frequency	AUC	Precision	Recall	F1
**Healthy Vs. DRFE**	**All**	**7.5-13.5 Hz**	0.77 (0.02)	71.42 (2.69)	73.96 (4.31)	72.57 (2.20)
**Healthy Vs. DRFE**	**Frontal, Temporal**	**7.5-13.5 Hz**	0.78 (0.04)	71.79 (5.39)	76.25 (10.50)	73.33 (4.06)
**Healthy Vs. DRFE**	**Frontal, Temporal**	**10.5-13.5 Hz**	0.83 (0.02)	79.41 (3.65)	73.54 (3.55)	76.24 (1.49)
**Seizure Focus**	**All**	**7.5-13.5 Hz**	0.68 (0.00)	72.73 (0.00)	85.71 (0.00)	78.69 (0.00)
**Seizure Focus**	**Frontal, Temporal**	**7.5-13.5 Hz**	0.73 (0.01)	74.19 (2.81)	93.21 (6.83)	82.39 (1.45)
**Seizure Focus**	**Frontal, Temporal**	**10.5-13.5 Hz**	0.77 (0.00)	80.00 (0.00)	85.71 (0.00)	82.76 (0.00)

Epilepsy associated disruptions in the alpha rhythm are significant when controlled for age, acquisition system differences, and antiepileptic medications

Finally, we sought to distinguish the contribution of chronic drug resistant epilepsy in the observed spectral changes of the alpha rhythm from the contributions of age and antiepileptic drugs (AEDs). In these evaluations, we calculated probability-of-normality estimates using the normalized alpha power features extracted from all brain regions and both low and high alpha frequency bands.

For Review Only

Effects of aging vs DRFE: In this analysis, we used the alpha features of healthy-young individuals (age: 20–35, N=97) for characterizing normal brain function based on CDFs. Then, using those CDFs, we computed the window-level probability-of-normality estimates (as described in methods) for healthy-old individuals (age: 59–77, N=47) and DRFE patients (N=48). Figure 6A illustrates the results of multiple comparisons performed on those probability-of-normality values between the different groups. Note that we used the data of healthy-young individuals as a reference in this analysis. We found that the differences between young and old healthy individuals, and the differences between healthy and DRFE individuals, were both significant ($p<0.01$). We also observed that the mean window-level probability-of-normality of DRFE patients was significantly lower than the means of other two groups.

Effects of acquisition system differences vs DRFE: In this analysis, we performed a three-way comparison between the healthy (N=144), PNES (N=104), and DRFE (N=48) populations. Note that the EEGs of PNES and DRFE patients were recorded using the same acquisition system (XLTEK, Inc.). Figure 6B illustrates the results of multiple comparisons performed on those probability-of-normality values between the different groups. Consistent with our hypothesis we we found difference between PNES and DRFE ($p<0.01$). Interestingly, we also found a difference between PNES and normal controls ($p<0.01$). We also observed that the mean window-level probability-of-normality of PNES patients was significantly lower than those of healthy individuals, and the mean probability-of-normality of DRFE patients was significantly lower than the means of both healthy and PNES groups.

Effects of AEDs vs DRFE: In this analysis, we used the alpha features of all healthy individuals (N=144) for characterizing normal brain function. Then, using those CDFs, we computed the window-level probability-of-normality values for two groups of DRFE patients: patients not taking any AEDs (N=5) and patients taking AEDs (N=43). Figure 6C illustrates the results multiple comparisons performed on those probability-of-normality estimates between the different groups. Like all previous analyses, we used the data of healthy individuals as a reference in this analysis. We found that the difference between DRFE individuals based on whether or not they took AEDs was significant ($p<0.05$). However, the mean probability-of-normality of DRFE individuals was still lower than that of the healthy individuals, regardless of whether or not they took AEDs.

Comparison between major AEDs: In this analysis, we divided the DRFE population based on the consumption of two AEDs: levetiracetam (N=25) or lamotrigine (N=19). We computed the window-level probability-of-normality values for those two groups of DRFE patients based on the CDFs obtained from the healthy population. Figure 6D illustrates the results multiple comparisons performed on those probability-of-normality estimates between the different groups. We found that the difference between DRFE individuals based on whether they took levetiracetam or lamotrigine was not significant ($p=0.25$).

For Review Only

Discussion

Main contribution of the study

In this study, we investigated whether visually classified normal EEGs of patients with epilepsy contain subtle abnormalities that may have diagnostic and clinical value. We conducted this retrospective study using scalp EEG recordings of 48 patients with drug-resistant focal epilepsy that were visually classified as normal EEG by expert review and the scalp EEGs of 144 age-matched healthy individuals. We extracted alpha power-related measures from eyes-closed (EC) segments in the EEGs of the healthy population and used them to represent normal brain activity. We then analyzed how the same alpha power-related measures in the DRFE population differed from in the healthy controls. Our analyses indicated that a) alpha power is significantly reduced in DRFE compared to healthy controls, and b) alpha power of right-handed DRFE patients with left hemispheric seizures is significantly lower compared to those with right hemispheric seizures. We also utilized these findings in a classification framework to classify 1) whether an EEG was recorded from an epilepsy patient, and 2) if so, the seizure generating side of the patient's brain, by using EEG recordings that do not contain any epileptiform activity. A ten-fold cross-validation approach achieved mean AUC values of 0.83 and 0.77 for the respective classification tasks (when high-alpha features from frontal-temporal regions were used). These findings suggest that EEG measures representing normal brain function can help in the diagnosis of epilepsy even when the EEG is free of epileptiform activity. This finding is significant because the ability to diagnose epilepsy at the earliest possible time can prevent significant delays in treatment and can support more efficient triage of patients to costly in-hospital monitoring studies. In that context, our study presents a promising research direction in the treatment of epilepsy.

Spatial and spectral patterns of the alpha abnormalities in DRFE

Our results showed significant abnormalities in the normalized spectral power of the alpha rhythm in frontal and temporal regions of DRFE patients (Figure 4A and 4C). This observation was further highlighted in the classification tasks; we found that using the alpha features extracted from frontal and temporal regions provided marginally better classification performances compared to using the same features extracted from all brain regions. These observations suggest that that the alpha abnormalities in DRFE are of focal nature and display frontal spreading. We surmise that such characteristic changes the alpha rhythm could have been modulated by the specific epilepsy syndrome because the majority of the DRFE patients (38 out of 48) had frontal or temporal seizure onset. Prior studies have also reported similar findings indicating frontal spread of alpha-rhythm alterations in focal epilepsy and suggested that this effect could be commensurate with the extent of cortico-thalamic dysfunction [17].

Our findings also agree with commonly known slowing of the alpha rhythm observed in the presence of neurological diseases including epilepsy [17]. Headplots shown in Figure 4A and 4C indicate that the differences between healthy and DRFE populations and the differences due to the side of seizure focus in the DRFE population are both stronger in the high alpha band (10.5-13.5 Hz) compared to low alpha band (7.5-10.5 Hz), based on the Wasserstein distance between CDFs. This finding was also signified in the classification performances; we found that using the spectral

For Review Only

power features extracted from high alpha band provided better classification performances in both the classification tasks compared to using the using the entire alpha band.

Analysis of confounders

EEG spectral abnormalities related to neurological diseases are typically confounded by age-related changes and changes induced by certain medications [15]. In this study, we showed that the alpha abnormalities we observed in the normal EEGs of DRFE patients were significant when compared with changes related to aging and AEDs, using a multiple comparison approach (Figure 6A and 6C). This finding suggests that the EEGs which were determined to be normal based on visual review, in fact, contain strong pathological correlates of DRFE that may be sufficient to support clinical use. However, we note that our analysis comparing the effects AEDs can benefit from additional samples to increase the statistical power of the finding.

Another potential confounder due to our study design is EEG acquisition system/environment differences. The EEGs of two main populations in this study, healthy control and DRFE, were acquired using different systems under different conditions. We utilized the EEG data of PNES patients, recorded using the same acquisition settings as DRFE patients, as the control dataset to study the contribution of this confounder. Our analysis (Figure 6B) showed that the decline in normal brain function seen in the DRFE population is significantly larger than that of the PNES patients. This observation, along with the fact that PNES is a psychiatric condition without known EEG correlate, suggests that the pathological differences between the healthy and DRFE populations are significant compared to the differences between acquisition systems.

Methodological contributions

We developed a probabilistic approach to characterize normal brain function using EEG features based on the alpha rhythm extracted from eight bipolar channels. This involves computing the CDFs of log-transformed alpha power features in the individual channels of 10-second EEG windows and using those CDFs to assign a window-level probability-of-normality estimate to new windows. We combined the individual channel-based values to obtain a combined probability-of-normality estimate for each window. Furthermore, we applied a maximum likelihood approach to aggregate the window-level probability-of-normality values of a participant's entire EEG recording to obtain probability-of-normality estimate for the participant. These values were then used for classification purposes. Our approach can be considered as an anomaly detection technique wherein we estimate whether a new sample is anomalous compared to the reference population. An advantage of this approach is that it can be developed using the data of the reference population alone without requiring data from anomalous samples. Furthermore, our approach presents a general paradigm for EEG-based anomaly detection, which can be beneficial for other EEG applications such as seizure forecasting [35]. In addition, our approach to characterize the health of brain function using the alpha rhythm can form the basis for the growing area of research on EEG and brain health [36] and can be used to study a variety of other neurological conditions.

For Review Only

Study limitations and future work

Our participants were limited to healthy individuals and DRFE and PNES patients. A normal EEG could be recorded from a patient with any number of other neurologic or psychiatric diseases. A population-level study including EEGs from a heterogeneous sample is necessary to accurately evaluate the clinical utility of our findings. Specifically in the context of epilepsy, the abilities to differentiate 1) drug-resistant epilepsy patients from drug-responsive epilepsy patients, and 2) focal epilepsy patients from generalized epilepsy patients, would be potentially very useful because they would allow clinicians to individualize treatments based on disease subtype.

Our analysis relied on expert EEG annotations regarding EC and EO conditions, awake and sleep, and bad channels. Such annotations are time consuming, costly, susceptible to human error, and clearly not scalable. Fully automated approaches that can analyze raw EEG data without requiring expert annotations of specific events can enable large scale studies, eliminate reviewer biases, and identify novel EEG features and advance scientific knowledge. Such automated approaches may also augment the visual review of epileptologists by providing focused inputs and help reduce physician burnout [37].

Another limitation of our study is that the EEGs of two populations, healthy and DRFE, were acquired using different systems under different conditions. To address this limitation as best we could, a) we undertook the same preprocessing steps for both the EEG datasets, and b) we used the fraction of alpha power within the wideband of 0.5-40 Hz to mask any subject-specific differences in total signal power. In addition, we also demonstrated using a population of PNES patients, that the differences between healthy and DRFE populations are not entirely due to acquisition system/environment differences. However, EEGs of both controls and patients recorded using the same acquisition system are necessary to confirm our results without this confounder. Our future efforts will investigate this possibility.

Conclusion

EEG-based diagnosis of epilepsy, which is the gold-standard approach, relies on visual identification of epileptiform activity. However, epileptiform activity may not be recorded in a short EEG recording session, and that can cause delays in the delivery of clinical care. Unfortunately, such scenarios are common in the clinic; approximately 50% of the EEGs recorded from patients with seizures are deemed normal based on expert visual review. In this study, we investigated the possibilities of diagnosing DRFE and lateralizing seizure focus based on normal EEGs using a semi-automated approach. Our results support the hypothesis that EEG-based measures of normal brain function, based on the alpha rhythm can help diagnose DRFE and lateralize seizure focus when an EEG does not contain any epileptiform activity, recorded seizures, or other non-specific abnormalities. Based on these findings, we further hypothesize that such findings in a normal EEG can suggest a higher pre-test probability for epilepsy when an individual is screened for epilepsy for the first time. In addition, our findings also suggest that automated analyses of scalp EEG can help in developing scalable and cost-effective approaches for advancing the current state of clinical electrophysiology. However, prospective studies and addressing the identified limitations of our work are necessary fully understand the clinical value of these hypotheses. Going forward, our efforts will focus on expanding the study to population-level

For Review Only

datasets including EEGs from heterogeneous samples, developing fully automated methods, and addressing systemic biases introduced by EEG acquisition systems.

Acknowledgements: We thank Kay Allen and Betty Baudoin for their help in data collection. This research was partly supported by a Mayo Clinic and Illinois Alliance Fellowship for Technology-based Healthcare Research, National Institute of Health grants NINDS-R01-NS92882, and NINDS-UH3-NS095495, National Science Foundation grants CNS-1337732 and CNS-1624790, IBM faculty award, Mayo Clinic Discovery Translation Grant, National Institutes of Health (R01-NS063039, R01-NS078136), and institutional resources for research by Czech Technical University in Prague, Czech Republic, ALISI – NPU (LO1212), VES15 II – LH15047.

Competing interests: Authors declare no competing interests.

References

1. Chen, Z., et al., *Treatment Outcomes in Patients With Newly Diagnosed Epilepsy Treated With Established and New Antiepileptic Drugs: A 30-Year Longitudinal Cohort Study.* JAMA Neurol, 2018. **75**(3): p. 279-286.
2. Uldall, P., et al., *The misdiagnosis of epilepsy in children admitted to a tertiary epilepsy centre with paroxysmal events.* Arch Dis Child, 2006. **91**(3): p. 219-21.
3. Binnie, C.D. and H. Stefan, *Modern electroencephalography: its role in epilepsy management.* Clin Neurophysiol, 1999. **110**(10): p. 1671-97.
4. Salinsky, M., R. Kanter, and R.M. Dasheiff, *Effectiveness of multiple EEGs in supporting the diagnosis of epilepsy: an operational curve.* Epilepsia, 1987. **28**(4): p. 331-4.
5. Bouma, H.K., et al., *The diagnostic accuracy of routine electroencephalography after a first unprovoked seizure.* European Journal of Neurology, 2016. **23**(3): p. 455-463.
6. Kwan, P., S.C. Schachter, and M.J. Brodie, *CURRENT CONCEPTS Drug-Resistant Epilepsy.* New England Journal of Medicine, 2011. **365**(10): p. 919-926.
7. Pillai, J. and M.R. Sperling, *Interictal EEG and the diagnosis of epilepsy.* Epilepsia, 2006. **47 Suppl 1**: p. 14-22.
8. Chen, T., et al., *The value of 24-hour video-EEG in evaluating recurrence risk following a first unprovoked seizure: A prospective study.* Seizure, 2016. **40**: p. 46-51.
9. Hauser, W.A., et al., *Seizure recurrence after a first unprovoked seizure.* N Engl J Med, 1982. **307**(9): p. 522-8.
10. Tao, J.X., et al., *Intracranial EEG substrates of scalp EEG interictal spikes.* Epilepsia, 2005. **46**(5): p. 669-76.
11. Ebersole, J.S. and S.V. Pacia, *Localization of temporal lobe foci by ictal EEG patterns.* Epilepsia, 1996. **37**(4): p. 386-99.
12. Ebersole, J.S. and R.F. Leroy, *Evaluation of ambulatory cassette EEG monitoring: III. Diagnostic accuracy compared to intensive inpatient EEG monitoring.* Neurology, 1983. **33**(7): p. 853-60.
13. Engel, J., Jr., et al., *Pathological findings underlying focal temporal lobe hypometabolism in partial epilepsy.* Ann Neurol, 1982. **12**(6): p. 518-28.
14. Liu, S. and J. Parvizi, *Cognitive refractory state caused by spontaneous epileptic high-frequency oscillations in the human brain.* Sci Transl Med, 2019. **11**(514).

For Review Only

15. Brown, E.N. and P.L. Purdon, *The aging brain and anesthesia.* Curr Opin Anaesthesiol, 2013. **26**(4): p. 414-9.
16. Halgren, M., et al., *The generation and propagation of the human alpha rhythm.* Proc Natl Acad Sci U S A, 2019. **116**(47): p. 23772-23782.
17. Abela, E., et al., *Slower alpha rhythm associates with poorer seizure control in epilepsy.* Ann Clin Transl Neurol, 2019. **6**(2): p. 333-343.
18. Knyazeva, M.G., et al., *Aging of human alpha rhythm.* Neurobiol Aging, 2018. **69**: p. 261-273.
19. Smith, S.J., *EEG in the diagnosis, classification, and management of patients with epilepsy.* J Neurol Neurosurg Psychiatry, 2005. **76 Suppl 2**: p. ii2-7.
20. Hyun, J., M.J. Baik, and U.G. Kang, *Effects of Psychotropic Drugs on Quantitative EEG among Patients with Schizophrenia-spectrum Disorders.* Clin Psychopharmacol Neurosci, 2011. **9**(2): p. 78-85.
21. Magalhaes, J.C., et al., *The influence of levetiracetam in cognitive performance in healthy individuals: neuropsychological, behavioral and electrophysiological approach.* Clin Psychopharmacol Neurosci, 2015. **13**(1): p. 83-93.
22. Meisel, C., *Antiepileptic drugs induce subcritical dynamics in human cortical networks.* Proc Natl Acad Sci U S A, 2020. **117**(20): p. 11118-11125.
23. Waldman, Z.J., et al., *Ripple oscillations in the left temporal neocortex are associated with impaired verbal episodic memory encoding.* Epilepsy Behav, 2018. **88**: p. 33-40.
24. Babayan, A., et al., *A mind-brain-body dataset of MRI, EEG, cognition, emotion, and peripheral physiology in young and old adults.* Sci Data, 2019. **6**: p. 180308.
25. Oostenveld, R. and P. Praamstra, *The five percent electrode system for high-resolution EEG and ERP measurements.* Clin Neurophysiol, 2001. **112**(4): p. 713-9.
26. Klem, G.H., et al., *The ten-twenty electrode system of the International Federation. The International Federation of Clinical Neurophysiology.* Electroencephalogr Clin Neurophysiol Suppl, 1999. **52**: p. 3-6.
27. Corballis, M.C., *Left brain, right brain: facts and fantasies.* PLoS Biol, 2014. **12**(1): p. e1001767.
28. Delorme, A. and S. Makeig, *EEGLAB: an open source toolbox for analysis of single-trial EEG dynamics including independent component analysis.* J Neurosci Methods, 2004. **134**(1): p. 9-21.
29. Palmer, J.A.a.K.-D., Ken and Makeig, Scott, *AMICA: An adaptive mixture of independent component analyzers with shared components*. 2012, University of California San Diego.
30. Bokil, H., et al., *Chronux: a platform for analyzing neural signals.* J Neurosci Methods, 2010. **192**(1): p. 146-51.
31. Ling, H. and K. Okada, *An efficient Earth Mover's Distance algorithm for robust histogram comparison.* IEEE Trans Pattern Anal Mach Intell, 2007. **29**(5): p. 840-53.
32. Gibbons, J.D. and S. Chakraborti, *Nonparametric statistical inference*. 5th ed. Statistics, textbooks & monographs. 2011, Boca Raton: Taylor & Francis. xx, 630 p.
33. Saville, D.J., *Basic statistics and the inconsistency of multiple comparison procedures.* Can J Exp Psychol, 2003. **57**(3): p. 167-75.
34. Bettinger, R., *Cost-sensitive classifier selection using the ROC convex hull method.* SAS Institute, 2003: p. 1-12.

For Review Only

35. Varatharajah, Y., et al., *Seizure Forecasting and the Preictal State in Canine Epilepsy.* Int J Neural Syst, 2017. **27**(1): p. 1650046.
36. Miraglia, F., et al., *EEG characteristics in "eyes-open" versus "eyes-closed" conditions: Small-world network architecture in healthy aging and age-related brain degeneration.* Clin Neurophysiol, 2016. **127**(2): p. 1261-1268.
37. Verghese, A., N.H. Shah, and R.A. Harrington, *What This Computer Needs Is a Physician: Humanism and Artificial Intelligence.* JAMA, 2018. **319**(1): p. 19-20.

For Review Only

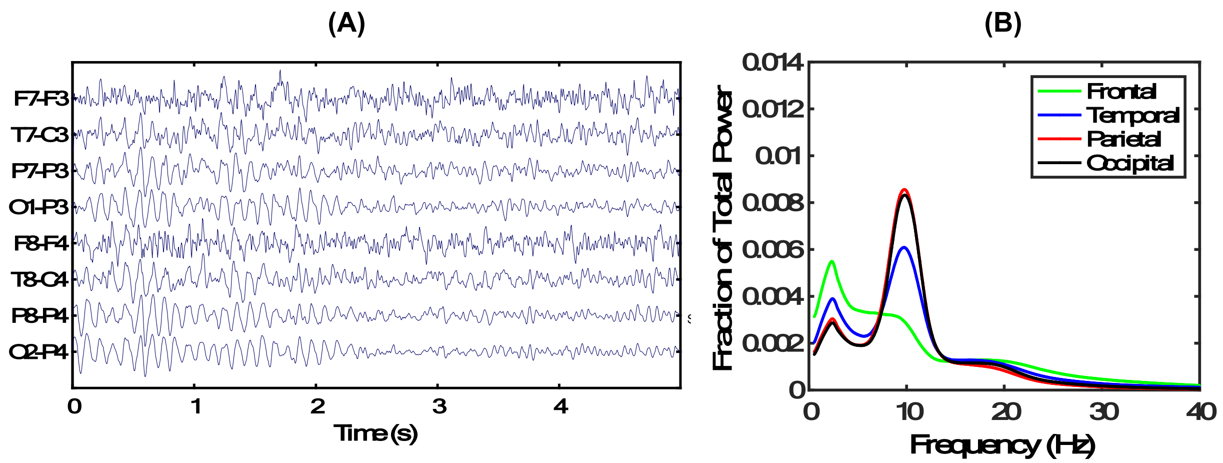

Figure 1: Extraction of spectral features. (A) illustrates a sample EC segment (length: 5-seconds) of a healthy participant's EEG. (B) illustrates normalized power spectrums of EC segments, where the solid line indicates average values calculated across all the healthy participants and the shaded areas indicate 95% confidence intervals.

For Review Only

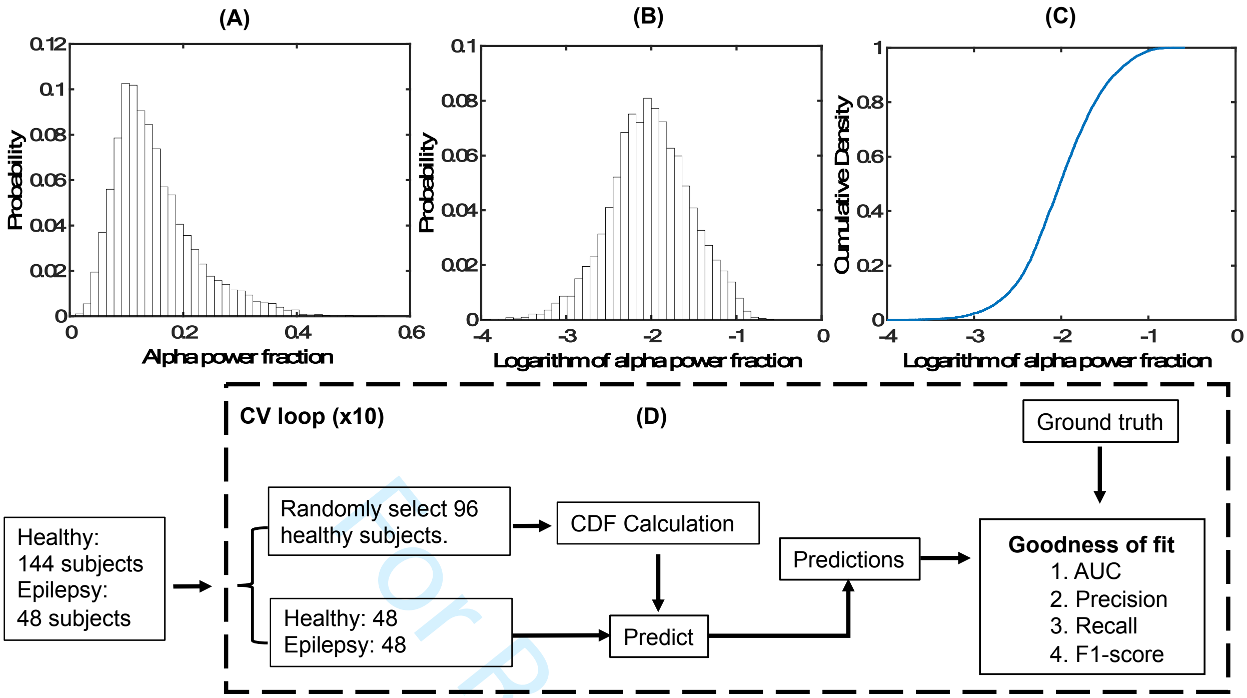

Figure 2: Characterizing normal brain function & classification framework. (A) Histogram of low-alpha (7.5-10.5 Hz) power fraction in (F7-F3) in the EC windows of healthy individuals. (B) and (C) illustrate the histogram and the cumulative density of the log transformation of the same features, respectively. (D) A random sample of 96 healthy participants are selected for characterizing normal brain function. The data of the rest (48 healthy and 48 DRFE) are utilized to evaluate the classification potential. Cumulative density functions of the 16 features representing alpha power fractions in the EC segments are computed. Window-level probability-of-normality estimates were aggregated to obtain participant level probabilities. Goodness of fit metrics were computed by comparing participant level probabilities with ground truth, separately for classifying a) healthy vs epilepsy and b) hemisphere with seizure focus. This procedure was repeated ten times to estimate average metrics and standard deviations.

For Review Only

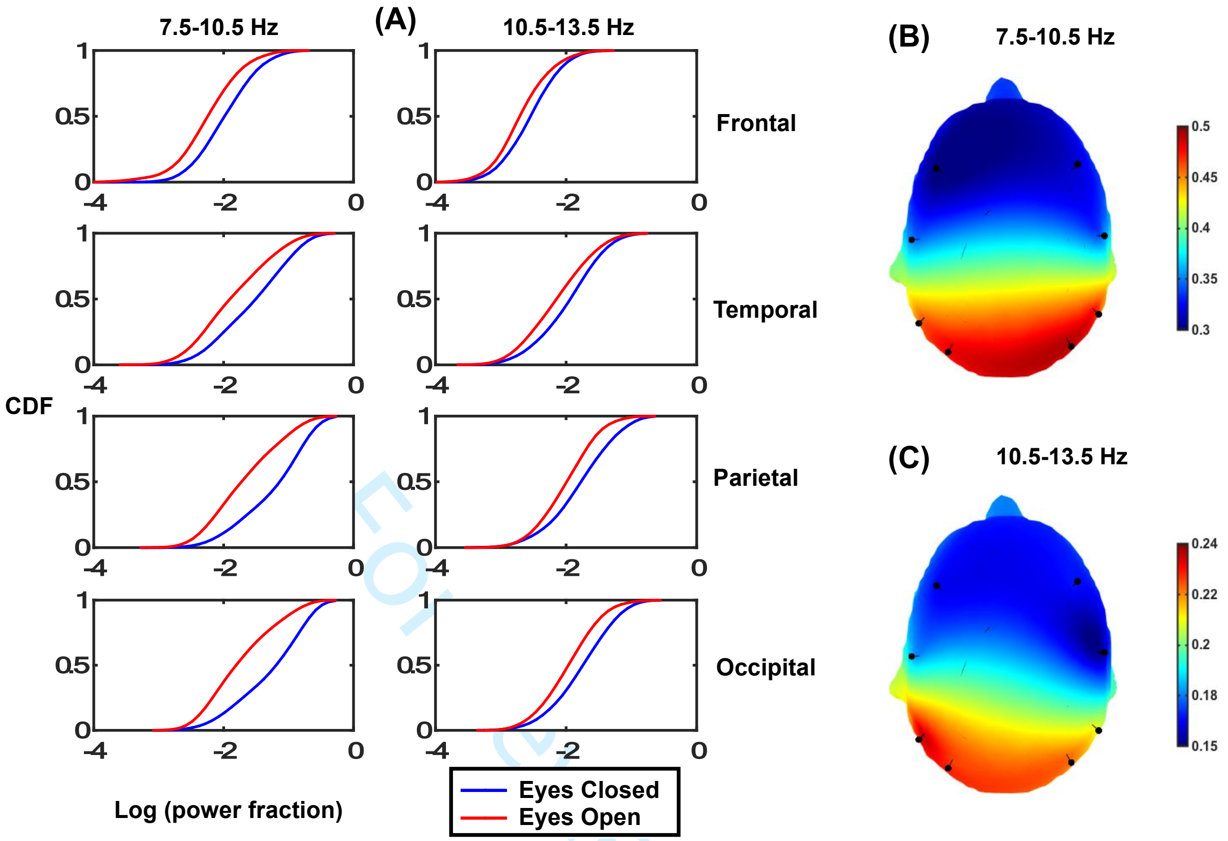

Figure 3: Characterizing normal brain function in the healthy population. (A) Cumulative density functions of log-transformed spectral power fractions in low-alpha (7.5-10.5 Hz) and high-alpha (10.5-13.5 Hz) bands, grouped based on eyes-closed and eyes-open conditions. Solid lines indicate average values across 10-second windows and shaded areas indicate 95% confidence intervals. Note that the window-level features were averaged between left and right hemispheres to generate the CDF plots. (B) and (C) are head plots illustrating the location-specific Wasserstein distances between the CDFs of EC and EO windows in low-alpha (7.5-10.5 Hz) and high-alpha (10.5-13.5 Hz) bands, respectively. The pins indicate approximate locations of the channels.

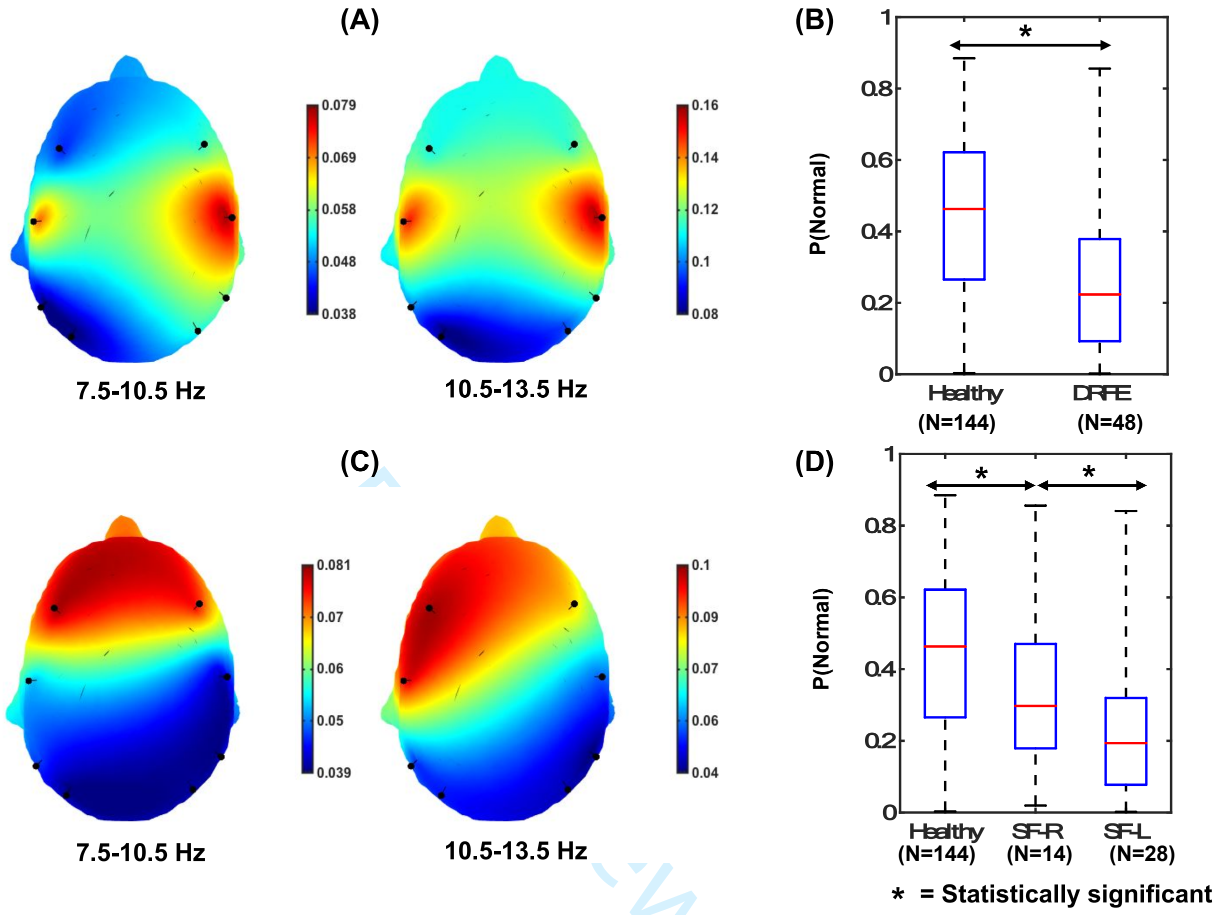

Figure 4: Disrupted normal brain function in the DRFE population. (A) Head plots illustrating the location specific Wasserstein distances between the CDFs of log-transformed alpha power features of healthy and DRFE individuals. (B) Boxplots of window-level probability-of-normality estimates for healthy and DRFE individuals. (C) Head plots illustrating the location specific Wasserstein distances between the CDFs of log-transformed alpha power features of right-handed DRFE individuals who had right-hemispheric seizures and those who had left-hemispheric seizures. (D) Boxplots of window-level probability of normality estimates for healthy and right-handed DRFE individuals where the DRFE individuals are further stratified based on the hemisphere generating seizures. In (B) and (D) the numbers N=n indicate the number of participants whose data were used in generating the boxplot.

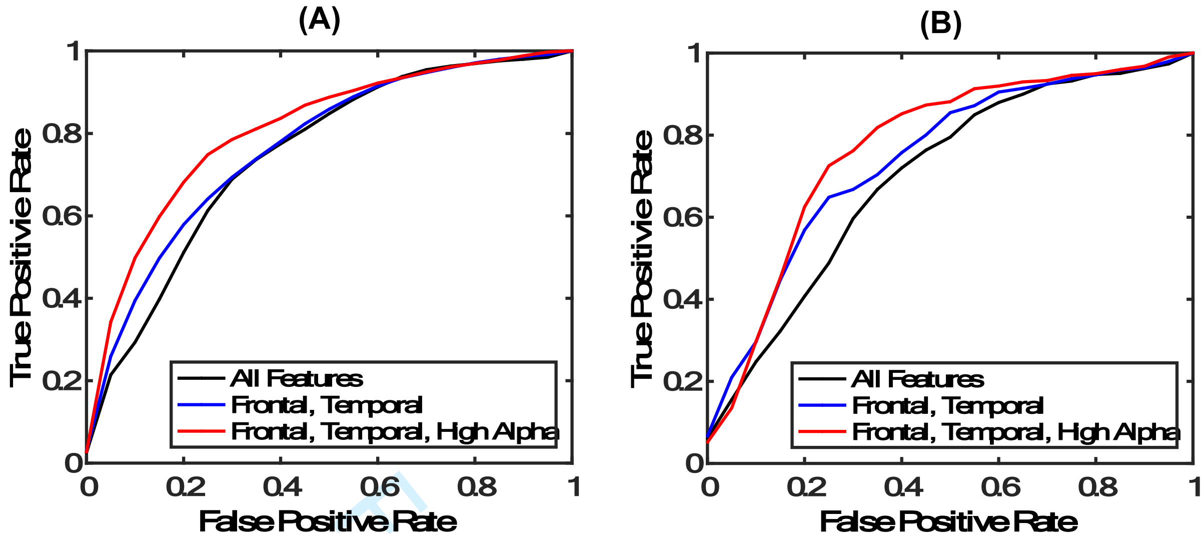

Figure 5: Receiver operating characteristic (ROC) curves for the two classification tasks. (A) ROC curves for classifying healthy individuals and DRFE patients. (B) ROC curves for classifying seizure generating side of the brain in right-handed DRFE patients. In both (A) and (B), red, blue, and black curves indicate classifications using features extracted in three ways: 1) from all regions and both alpha bands, 2) frontal-temporal regions and both alpha bands, and 3) frontal- temporal regions and high alpha band only, respectively. Furthermore, solid lines indicate average values obtained using the tenfold cross validation and shaded areas indicate 95% confidence intervals.

For Review Only

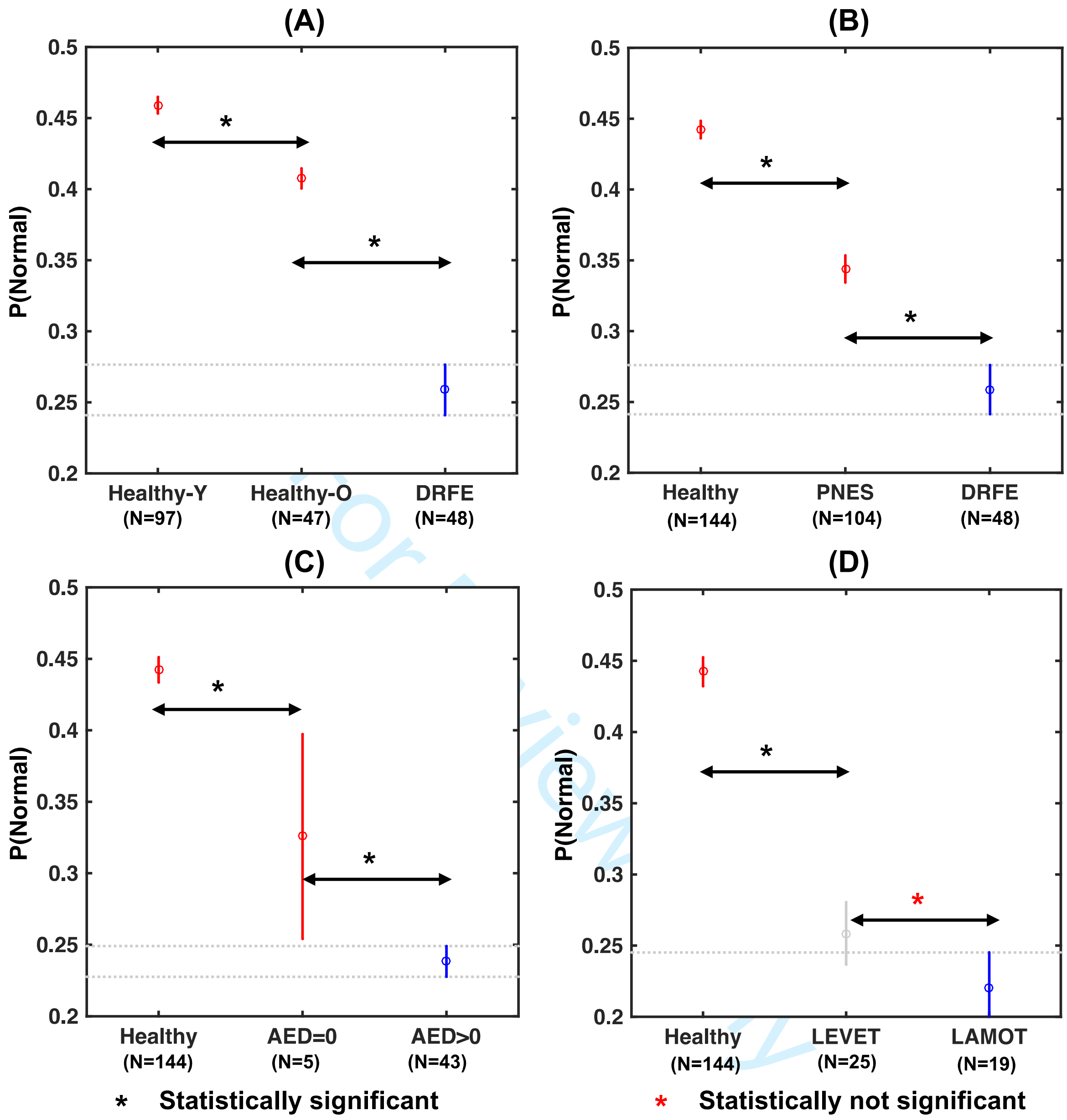

Figure 6: Multiple comparisons analyzing the contributions of age, acquisition systems, and antiepileptic drugs (AEDs). Figures show the point estimates and comparison intervals of mean probability of normality values. (A) A comparison between healthy and DRFE individuals where the healthy individuals are stratified based on the age-group: Young (Healthy-Y): 20–35, and Old (Healthy-O): 59–77). (B) A comparison between healthy individuals and patients with PNES and DRFE, where the EEGs of both groups of patients were acquired using the same acquisition system. (C) A comparison between healthy and DRFE individuals where the DRFE individuals are stratified based on the number of AEDs consumed at the time of EEG. (D) A comparison between healthy and DRFE individuals where the DRFE individuals are stratified based on the primary AEDs consumed at the time of EEG: levetiracetam or lamotrigine. The numbers N=n indicate the number of participants whose data were used in the analyses.
