# Supplementary material for: Characterizing the electrophysiological abnormalities in visually reviewed normal EEGs of drug-resistant focal epilepsy patients
Source: Brain Commun. 2021 May 14;3(2):fcab102. doi: 10.1093/braincomms/fcab102 (PMC8196245; doi:10.1093/braincomms/fcab102)
Supplement: fcab102_Supplementary_Data [file fcab102_supplementary_data.zip › Supplementary_data.docx]

**Supplementary figure 1.** AED differences and arousal state differences. (A) A comparison between healthy and DRFE individuals where the DRFE individuals are stratified based on the primary AEDs consumed at the time of EEG: levetiracetam or lamotrigine. (B) and (C) Differences in the rate of eye blinks between healthy and DRFE individuals. (B) all potential blinks as identified by the software and (C) only those blinks that have R^2^ > 0.90 between upstrokes and downstrokes of the blinks.

**Supplementary figure 2.** ROC curves for classifying PNES patients taking AEDs vs. DRFE patients taking AEDs.

|  | **AUC** | **Precision** | **Recall** | **F1** |
| --- | --- | --- | --- | --- |
| **PNES vs. DRFE – All regions and features** | 0.62 (0.00) | 76.09 (0.00) | 79.55 (0.00) | 77.78 (0.00) |
| **PNES vs. DRFE – Frontal, Temporal** | 0.56 (0.00) | 64.91 (0.00) | 84.09 (0.00) | 73.27 (0.00) |
| **PNES vs. DRFE – Frontal, Temporal – high alpha only** | 0.60 (0.00) | 72.09 (0.00) | 70.45 (0.00) | 71.26 (0.00) |

**Supplementary table 3.** Goodness of fit metrics for classifying PNES patients taking AEDs vs. DRFE patients taking AEDs (note that the standard deviations are 0 because there is no variability in the classification performance).

**Supplementary figure 3.** Boxplots of window-level probability of normality estimates for healthy and right-handed DRFE individuals where the DRFE individuals are limited to those with temporal lobe seizure onset and further stratified based on the hemisphere generating seizures (SF-R-T: right temporal seizure focus and SF-L-T: left temporal seizure focus).
