# Supplementary material for: Characterizing the electrophysiological abnormalities in visually reviewed normal EEGs of drug-resistant focal epilepsy patients
Source: Brain Commun. 2021 May 14;3(2):fcab102. doi: 10.1093/braincomms/fcab102 (PMC8196245; doi:10.1093/braincomms/fcab102)
Supplement: fcab102_Supplementary_Data [file Revision 1 – Decision Letter .pdf › Revision 1 – Decision Letter .pdf]

Revision 1 – Decision Letter

08-Apr-2021

Dear Dr. Varatharajah,

It is a pleasure to accept your manuscript entitled "Characterizing the Electrophysiological Abnormalities in Visually-reviewed Normal EEGs of Drug-Resistant Focal Epilepsy Patients" for publication in Brain Communications. Any comments made by the reviewer(s) are included below.

The next step is for your paper to go through "First Look". We will contact you again shortly if any final files or formatting changes are required for publication or if we have any queries.

If no changes are needed, your submission will be transferred to our Production Department and you will hear from the Production Editor in due course.

Video abstracts - Brain Communications encourages authors to provide a video abstract to accompany their article. These are hosted on the Brain/Brain Communications YouTube channel. If you are interested in producing a 5-min video abstract to promote your article, then please contact the Editorial Office (brcoms.editorialoffice@oup.com) for further details and guidelines.

Thank you very much for your contribution. On behalf of the Editors of Brain Communications, we look forward to your future contributions to the Journal.

Yours sincerely,

Professor Richard Chin
Associate Editor
Brain Communications

Reviewer(s)' Comments to Author:

Reviewer: 2

Comments to the Author
The authors have addressed my comments, and I recommend the manuscript for publication as it is.

Reviewer: 1

Comments to the Author
The authors have extensively revised the text and added analyses that help address potential confounding variables, thereby significantly increasing confidence in the main results. The shading around lines in Figs. 3 and 5 still does not appear in my PDF but I assume this will be addressed at time of production. I have no further concerns.

Date Sent:
