# Supplementary material for: Characterizing the electrophysiological abnormalities in visually reviewed normal EEGs of drug-resistant focal epilepsy patients
Source: Brain Commun. 2021 May 14;3(2):fcab102. doi: 10.1093/braincomms/fcab102 (PMC8196245; doi:10.1093/braincomms/fcab102)
Supplement: fcab102_Supplementary_Data [file Original Submission – Decision Letter (1).pdf › Original Submission – Decision Letter (1).pdf]

Original Submission – Decision Letter

01-Mar-2021

Dear Dr. Varatharajah,

Thank you for your recent submission BRAINCOM-2021-054 entitled "Characterizing the Electrophysiological Abnormalities in Visually-reviewed Normal EEGs of Drug-Resistant Focal Epilepsy Patients" to Brain Communications.

Your manuscript has been reviewed, and while the reviewers and editorial team appreciate many aspects of the paper, they raise some concerns that you will need to address in order for the paper to be considered for publication.

If you can address these concerns, I invite you to submit a revised manuscript and a response to each of the reviewers' comments. The reviewer(s)' comments are included at the bottom of this message.

In addition to the reviewers' comments, please note our editorial comments added below to comply with our requirements for presentation of scientific data at Brain Communications.

Please format your revised manuscript according to our Instructions to Authors https://academic.oup.com/braincomms/pages/General_Instructions – a summary of key points is attached to help with this.

To revise your manuscript, click the link below or log into https://mc.manuscriptcentral.com/braincom and enter your Author Centre, where you will find your manuscript title listed under "Manuscripts with Decisions." Under "Actions," click on "Create a Revision." Your manuscript number has been appended to denote a revision.

*** PLEASE NOTE: This is a two-step process. After clicking on the link, you will be directed to a webpage to confirm. ***

https://mc.manuscriptcentral.com/braincom?URL_MASK=1f77b6ae7af54c649a82b1898a999159

Please upload a revised manuscript with changes tracked with track changes or in coloured text.

When submitting your revised manuscript, you will be asked to respond to the reviewer(s) comments. Please be as specific as possible in your response to all of the comments.

The editorial team at Brain Communications aims to increase transparency in scientific publishing and to demonstrate the value that peer review brings to the scientific process. Please therefore consider whether you are willing to have reviewer(s)' comments on your paper and your response published as supplementary data with the paper if it is accepted. This is entirely optional and will only be done if both the authors and reviewers agree. While submitting your revision there will be an opportunity to review or update the answer you gave previously, if necessary.

IMPORTANT: Your original files are available to you when you upload your revised manuscript. Please delete any redundant files before completing the submission.

Because we are trying to facilitate timely publication of manuscripts submitted to Brain Communications, we would appreciate your revised manuscript as soon as possible. If it is not possible for you to resubmit within 3 months, the option to submit a revision will expire, and we would then consider your revised paper as a new submission. However, if you need a little more time, please get in touch and we can extend this deadline for you.

Once again, thank you for submitting your manuscript to Brain Communications and I look forward to receiving your revision.

Yours sincerely,

Prof. Richard Chin
Editor, Brain Communications
r.chin@ed.ac.uk

Reviewer(s)' Comments to Author:

Reviewer: 1

Comments to the Author
Summary:
Scalp electroencephalography (EEG) is a critical tool in the diagnosis of epilepsy, but, despite its reasonably high specificity, the sensitivity of scalp EEG for epilepsy is poor. Thus, many patients with epilepsy have a normal scalp EEG, which can lead to delayed diagnosis and treatment. In this report, Varatharajah and colleagues analyzed routine scalp EEGs that had been interpreted by expert readers as normal in people with drug-resistant focal epilepsy (DRFE) and in healthy controls. They found that, although all EEGs had been read as normal, normalized spectral alpha power was lower in the EEGs of people with DRFE than in healthy individuals, and, within people with DRFE who are right-handed, that alpha power was lower when seizures arose from the dominant (left) hemisphere. They develop classifier algorithms to distinguish these groups based on alpha power, and best areas under the receiver operating characteristic curves (ROC AUCs) were 0.83 (healthy vs. DRFE) and 0.77 (seizure lateralization). They perform control analyses for potential confounders, including age, anti-seizure medications, and EEG recording system (healthy controls were from a publicly available dataset (LEMON) recorded under different conditions than DRFE patients), with the latter facilitated by normal EEGs from patients with psychogenic non-epileptic seizures (PNES) who were recorded under similar conditions as DRFE. The authors conclude that screening for alpha power in normal EEGs could increase pre-test probability for epilepsy in some individuals who have a normal EEG, thereby motivating additional workup and enabling earlier diagnosis and treatment.

Significance:
The insensitivity of routine scalp EEG for diagnosing epilepsy is a significant clinical challenge, one that is typically overcome by repeated and/or longer recordings. Given the ubiquity of EEG, new methods to extract reliable prognostic information from routine scalp recordings would be of broad interest to epileptologists and general neurologists.

Strengths:
The manuscript is generally clear and well-written. I particularly appreciated the detailed Methods section, which is easy to follow, and I commend the authors for their earnest efforts to control for several potential confounders (i.e. age, anti-seizure medications, recording system differences). Conclusions are not overstated, and some study limitations are appropriately acknowledged. The figures are generally clear and well-constructed (see a few minor comments below).

Overall, I enjoyed reading this manuscript. However, I have several major and minor concerns, which, if adequately addressed, would increase my enthusiasm for the paper:

Major Concerns:
1. Effects of arousal state: A potential problem with using alpha power as an EEG biomarker is that it is heavily dependent on a patient's arousal state. For example, alpha power is usually maximal in the eyes closed (EC) condition when transitioning from alertness to relaxed wakefulness, but alpha activity usually decreases during transition to the drowsy state, in which theta frequencies predominate. It is hard for us to know whether the EC segments analyzed reflect relaxed wakefulness or a drowsy state. Thus, it is possible that the main effects observed by the authors (decreased fractional alpha power in patients with DRFE) reflects systematic differences in arousal states captured by EEG in patients with DRFE compared with healthy controls. Indeed, patients with DRFE are probably much more likely to be drowsy during their EEG (owing to a variety of factors: sedating effects of anti-seizure medications, sleep fragmentation, recent seizures, etc.) than healthy controls from the LEMON dataset, who were very likely to be briskly awake because they had very brief (16-min) recordings and "were seated in front of a computer screen and asked to stay awake while fixating eyes on a black cross presented on a white background (during the eyes-open sessions)" (Babayan et al., https://www.nature.com/articles/sdata2018308).

Of note, the apparent effects of seizure lateralization on alpha power could potentially help allay concerns about systematic differences in arousal state in people with DRFE vs. healthy controls, but the significant imbalance in sample sizes (28L, 14R; Fig. 4D) makes apparent differences hard to interpret (the supplemental Excel file is helpful, but it would be even better if the breakdown of frontal/temporal seizure localization was quantified by left vs. right hemispheric onset, to ensure similar distribution, because these regions seem to be where the alpha power effects are most salient).

2. Effects of recording system: As the authors state, "PNES is a psychiatric condition without known EEG correlate". Thus, the sizeable difference in probability of normality ('P(normal)') values between Healthy controls and PNES patients (Fig. 6B), who were recorded with different EEG acquisition systems, is curious. The authors rightly point out that P(normal) values of patients with DRFE are still significantly lower than those with PNES (Fig. 6B), but this observation nevertheless suggests that recording system differences may account for much of the observed main effect. To their credit, the authors acknowledge in the Discussion that it would be cleaner to have controls and patients recorded with the same

acquisition system to eliminate this concern.

3. Effects of AEDs: The authors find that P(normal) values are lower for patients with DRFE who are not on AEDs than healthy controls, supporting their argument that their observations are not explained solely by medication effects. However, the fact that patients with DRFE who were on AEDs had an additional statistically-significant decrease in P(normal) compared to patients with DRFE who were not on AEDs (Fig. 6C) suggests that medications might account for some of the observed effects on alpha power. Indeed, if one mentally combines the left half of Fig. 6B with the right half of Fig. 6C, it seems that the observed Healthy vs. DRFE alpha power differences can be almost entirely accounted for by recording system differences and AED effects; i.e. perhaps the difference between Healthy and PNES is driven by recording system differences, and the difference between PNES and DRFE is driven by medication effects in the majority of DRFE patients who were taking AEDs?

Taken together, these concerns regarding potential confounding variables cast doubt on the validity of the authors' findings.

Minor Concerns:
1. Introduction, line 12: "…in patients determined to have DRE"; might be better to rephrase as "in patients found to have DRE" to avoid double-meaning of the word 'determined'

2. Introduction, "EEG is the primary method to diagnose epilepsy". Some readers might take umbrage with this statement, as epilepsy is a clinical diagnosis and History is usually the paramount diagnostic tool, particularly given the many limitations of EEG.

3. Methods: Could the authors please specify the criteria by which certain channels were deemed to be outliers?

4. Several figure legends refer to 'shaded areas' indicating confidence intervals, but these are not visible in the PDF I reviewed (unless the interval are extremely tight). Perhaps something got lost during file conversion?

5. Figs. 3 and 4: I assume the head plots are top down views (i.e. not radiographic convention), but labeling L and R might help avoid confusion. Also, the authors state that CDF plots were "nearly symmetric" but some asymmetry is apparent in Fig. 3C—could the authors please comment?

6. The Discussion should include some comments on envisioned clinical applications of a test with an AUC of 0.8, including ways it could be helpful (i.e. avoid delayed diagnosis/treatment, as currently mentioned) and also unhelpful (i.e. morbidity of unnecessary testing/treatment when an imperfect biomarker is falsely positive).

Thank you for the opportunity to review this interesting manuscript.

Reviewer: 2

Comments to the Author
This manuscript presents evidence for the reduction of alpha power (derived from resting-state EEG) in drug-resistant focal epilepsy, as well as significant differences of alpha power between patients with left and right hemispheric seizures. It is further demonstrated that alpha power has the potential to classify focal epilepsy as well as the hemisphere of seizure emergence. This study marks an important step towards optimising the use of standard EEG in the clinic.

The manuscript is well written, and all steps of the investigation can be easily followed. Nonetheless, I do have some reservations regarding the use of 'probability-of-normality' (PON). First of all, the abstract states that alpha power is reduced in DRFE etc, yet the manuscript only presents implicit evidence for this via the PON measure. The results would be much more tangible if the (averaged) alpha power fraction were used directly. One problem I see with the PON is that the necessary assumption that alpha power is reduced in epilepsy prevents to generalise the PON (e.g. a hypothetical disorder that shows an increase of alpha power would then show a higher PON than healthy controls). The use of the term PON can also be a bit confusing, as I first thought it refers to the likelihood that the log-transformed alpha power is normally distributed (Fig 2B). Additionally, the PON is roughly 0.45 in healthy controls; I would expect this to be close to 1.

Given the fact that the PON is based on monotone transformations (log, CDF) of the alpha power, and that the nonparametric tests (as well as the ROC) are rank based, I am convinced that the results would remain virtually unchanged if the alpha power between the test groups is compared directly. Otherwise, the use of an elaborate method such as the PON has to be better motivated, i.e. it has to be explained

what it yields that alpha power doesn’t.

Minor points / typos:

- In Fig 1B and Fig 5, shaded areas are missing

- labels and numbers in Figs 1- 5 appear to be squeezed

- p.3 l. 20/21: and in —> in

- p.7 l. 37: Furthremore —> Furthermore

- p. 7, Eq (2): N(i) has to be defined

- p.8 l.46: cruve —> curve

- p.13 l.16: from in —> from

- p.13 l.39: that that —> that

- p.15 l.54 fully —> to fully

- legend of Figs 4 & 6: N=n —> N

Reviewer: 3

Comments to the Author
This manuscript develops the recent interest in identification of features of apparently normal EEG that might identify the presence of a seizure disorder or epilepsy in patients. The translational potential of such approaches is high, due to the vast majority of clinically recorded EEGs in the epilepsy diagnosis pathway currently being viewed as normal and therefore clinically uninformative.

In general I think the findings presented in this manuscript are interesting, however I believe the authors could place their work in better context of the existing literature around interrogation of apparently normal EEG in the context of epilepsy, as well as consider some challenges relevant to group-level observations versus individual predictions, which are of importance for the ultimate translation of these findings. I expand on these comment below.

In this manuscript the authors state that “we hypothesized that even normal EEGs might contain subtle electrophysiological cues of epilepsy”. There is a substantial body of work that explores this and presents strong evidence for it, so I think it is not the best use of language to describe it as a hypothesis, particularly as it gives the implication that the authors themselves generated it, which is I am sure not the impression they sought to give. For example in the specific context of alpha power Larson et al Clin Neurophys 116, 2701 (2005) first identified a relationship between decrease in alpha power and people with epilepsy. Whilst they didn’t focus specifically on DRFE vs well-controlled, it is a clear extension of this work that the hypothesis would be “lower frequency” correlates with poorer likelihood of seizure freedom. I feel the authors should include reference to this manuscript and a discussion of how their work builds on its findings.

Now in their introduction, I feel it is important for the authors to give a more holistic overview of ongoing research that explores the relationship between apparently normal EEG and risk of epilepsy. For example, back in 2014 Chowdhury et al PLOS ONE 110136 demonstrated for the first time that network properties, as inferred from background EEG, were altered, both in people with generalized epilepsies as well as their first degree relatives. Of note to the present study is that the frequency band within which the EEG was filtered and these networks were inferred was termed “low alpha” defined as 6-9Hz. Building on this, the same group Schmidt et al 2014 PLOS Comp Biol 1003947 demonstrated that changes in these networks supported a dynamic adaptation to hypersynchrony, making it easier for seizures to emerge. Most recently, Woldman et al 2020 Sci Rep 10 7043 used a computer model to demonstrate how altered functional networks in these low frequency bands could support EEG-like activity that was either confined to a single hemisphere, or bilaterally spread. This provided evidence for network alteration being an enduring feature of seizures, and that it is possible to identify a focal or generalized seizure phenotype from apparently normal background EEG. Further work of relevance to support the use of background EEG in the context of seizure/epilepsy identification is that of Verhoeven et al 2017 NeuroImage Clinical 17, 10-15 who presented an algorithm for identifying cases of temporal lobe epilepsy from background EEG in the absence of interictal spikes. This study used source-based network reconstruction across 80 or so regions of interest and achieved approaching 90% accuracy in a small cohort of 40 subjects. Very recently Pegg et al 2021 Epilepsia 62, 492 considered the difference

between drug resistant and well controlled IGE cases, finding that properties of the inferred networks correlated strongly with seizure freedom or poor control. The authors may find a recent systematic review of graph measures in interictal data from IGE from the same group useful: Pegg et al 2020 Epilepsy Behav 107013.

In the methods, I could not understand the rationale for handedness to determine the "dominant" hemisphere, and why this would be important in terms of revealing the seizure generating propensity of the brain. For example, contrasting with the approach mentioned earlier of Woldman et al 2020? Could the authors expand a little as to why the dominant hemisphere is important, given there was a split of left and right sided epilepsies in the cohort?

The results are in general clearly presented, but again I feel it would be helpful for the reader if the authors were to describe the key differences between their current approach, and that of Larson et al mentioned earlier.

In their discussion, I feel the authors might like to give some consideration to potential limitations when it comes to the application of these findings at an individual level. Here I refer to Schmidt et al 2016 Epilepsia 57, e200. In this study the authors consider a cohort for people with IGE vs healthy controls and used a classifier to explore the performance of markers (including alpha power) previously identified as distinguishing between the cohorts at a group level. Alpha power (in the sense introduced by Larson) was shown to have 0 accuracy at an individual level, and so would not be an appropriate marker. The authors may wish to discuss their findings in the context of group level effects vs individual differences.

Finally, in their discussion of limitations the authors rightly raise the issue that they have compared people with epilepsy with health controls. However they may wish to frame this more in the context of diagnostic/prognostic biomarker studies, where a phase I/II study would be to demonstrate that a marker is effective, and that it can distinguish those with a condition, from those clearly without the condition. For example Yee et al 2018 Bioanalysis 10, 4155.

Editorial Office comments:

-Fig1b:please, use different line styles to identify the data groups.

-Please supply a graphical abstract. A graphical abstract is a visual representation of the central finding or methodology of the paper. It will be displayed in the table of contents. Authors should strive to make the graphical abstract informative, interesting, visually appealing, and straightforward (no caption needed, no abbreviations except gene names). Please upload as a separate high resolution image file (tiff preferred). The technical requirements for the graphical abstracts are:
  --size of the submitted image: 1200 pixels square at 300dpi
  --font of the text: Arial 12-16 point (smaller fonts will not be legible online)

-Please add an Abbreviated Summary (up to 50 words) of your paper that captures the main purpose and conclusions of the work. The summary should state the main results written in the third person ('[1st author surname] et al. report that …') and conclusion. This summary will be used in the contents list online and should not contain any abbreviations other than those on the accepted abbreviations list and accepted gene/protein names, making it accessible to the non-specialist. Please upload it as a Word document. Allowed abbreviations: AIDS; ANOVA; AMPA; ATP; A,T,C,G; CNS; CSF; CT; DNA; ECG; EEG; EMG; GABA; HIV; MRI; NMDA; PET; PCR; RNA.

- To comply with our ethical standards, the appropriate checklist must be included (see Reporting Guideline section within 'Instructions to authors').
