# Supplementary material for: Characterizing the electrophysiological abnormalities in visually reviewed normal EEGs of drug-resistant focal epilepsy patients
Source: Brain Commun. 2021 May 14;3(2):fcab102. doi: 10.1093/braincomms/fcab102 (PMC8196245; doi:10.1093/braincomms/fcab102)
Supplement: fcab102_Supplementary_Data [file Original Submission- Author's Response.pdf › Original Submission- Author's Response.pdf]

We appreciate the detailed reviews provided by the reviewers. We addressed each concern in the revised manuscript. In the following, we respond (black) to each of the reviewers' concerns (red).

Reviewer: 1

Comments to the Author

Strengths:
The manuscript is generally clear and well-written. I particularly appreciated the detailed Methods section, which is easy to follow, and I commend the authors for their earnest efforts to control for several potential confounders (i.e. age, anti-seizure medications, recording system differences). Conclusions are not overstated, and some study limitations are appropriately acknowledged. The figures are generally clear and well-constructed (see a few minor comments below).

Overall, I enjoyed reading this manuscript. However, I have several major and minor concerns, which, if adequately addressed, would increase my enthusiasm for the paper:

Thank you for the positive and constructive feedback. We performed several additional experiments to address the concerns and believe that the new results significantly strengthen our findings.

Major Concerns:
1. Effects of arousal state: A potential problem with using alpha power as an EEG biomarker is that it is heavily dependent on a patient's arousal state. For example, alpha power is usually maximal in the eyes closed (EC) condition when transitioning from alertness to relaxed wakefulness, but alpha activity usually decreases during transition to the drowsy state, in which theta frequencies predominate. It is hard for us to know whether the EC segments analyzed reflect relaxed wakefulness or a drowsy state. Thus, it is possible that the main effects observed by the authors (decreased fractional alpha power in patients with DRFE) reflects systematic differences in arousal states captured by EEG in patients with DRFE compared with healthy controls. Indeed, patients with DRFE are probably much more likely to be drowsy during their EEG (owing to a variety of factors: sedating effects of anti-seizure medications, sleep fragmentation, recent seizures, etc.) than healthy controls from the LEMON dataset, who were very likely to be briskly awake because they had very brief (16-min) recordings and "were seated in front of a computer screen and asked to stay awake while fixating eyes on a black cross presented on a white background (during the eyes-open sessions)" (Babayan et al., https://urldefense.com/v3/__https://www.nature.com/articles/sdata2018308__;!!DZ3fjg!qqX1ar0w1n97HEZT4keCaa8mauMYz4gHHjNDv0vaNILyFyAKWP38xI0yy-P3Q6Z_sLM$).

This is a very good point. To analyze this effect, we utilized the rate of eye blinks as a surrogate measure for drowsiness (Jung, Makeig et al. 1997, DiGirolamo, Patel et al. 2016) and compared the number of eye blinks per minute in the eyes open EEG segments of healthy and DRFE individuals. Eye blinks were counted using a well-recognized tool available in the EEGLAB software (Kleifges, Bigdely-Shamlo et al. 2017). Figure 6F shows a comparison between the rates of eye blinks of the two groups where the criterion that the upstrokes and downstrokes of the eye blinks were significantly correlated ($R^2 > 0.90$) was used to identify robust eye blinks. We found that the two patient populations studied here (epilepsy v.s. normals) were not different with respect to the rate of eye blinks (p=0.89). A comparison between all potential eye blinks is provided in Supplementary figure 1B, where the difference is still not significant (p=0.23).

Of note, the apparent effects of seizure lateralization on alpha power could potentially help allay concerns about systematic differences in arousal state in people with DRFE vs. healthy controls, but the significant imbalance in sample sizes (28L, 14R; Fig. 4D) makes apparent differences hard to interpret (the supplemental Excel file is helpful, but it would be even better if the breakdown of frontal/temporal seizure

localization was quantified by left vs. right hemispheric onset, to ensure similar distribution, because these regions seem to be where the alpha power effects are most salient).

We note that the majority of our patients had unilateral temporal lobe epilepsy (N=31) and a few had unilateral frontal onset (N=3). The rest of the patients had onsets in other regions, or bilateral onsets. We repeated the analysis in Figure 6D focusing only on unilateral temporal onset (shown in Supplementary figure 3) and our results remain unchanged. Furthermore, we agree with the reviewer that the two classes are imbalanced in this case. However, we refer to the classification analysis in Figure 5B and note that a classification performance substantially better than chance (AUC=0.77) in differentiating the side of seizure focus. In the machine learning literature, the AUC measure is considered a robust measure of classification performance despite any class imbalance present in the data (Liu, Wu et al. 2009).

2. Effects of recording system: As the authors state, "PNES is a psychiatric condition without known EEG correlate". Thus, the sizeable difference in probability of normality ('P(normal)') values between Healthy controls and PNES patients (Fig. 6B), who were recorded with different EEG acquisition systems, is curious. The authors rightly point out that P(normal) values of patients with DRFE are still significantly lower than those with PNES (Fig. 6B), but this observation nevertheless suggests that recording system differences may account for much of the observed main effect. To their credit, the authors acknowledge in the Discussion that it would be cleaner to have controls and patients recorded with the same acquisition system to eliminate this concern.

We appreciate the feedback and agree that the recording system differences may account for some of the effect. However, even assuming this is the case we note that the PNES patients cannot be treated as 'healthy' individuals. Specifically, we provide additional information on this population (Supplementary table 2 on PNES patients), where it is clear that these patients are on several psychoactive medications (antiepileptic drugs because of mood stabilizing properties of drugs in that class, antidepressants, and antipsychotics) at the time of EEG and have a range of psychiatric comorbidities, which themselves may alter baseline alpha (Kaiser, Gnjezda et al. 2018, Newson and Thiagarajan 2018). Apart from these, the PNES disease itself, although without known EEG features, can add to the difference between healthy and PNES.

3. Effects of AEDs: The authors find that P(normal) values are lower for patients with DRFE who are not on AEDs than healthy controls, supporting their argument that their observations are not explained solely by medication effects. However, the fact that patients with DRFE who were on AEDs had an additional statistically-significant decrease in P(normal) compared to patients with DRFE who were not on AEDs (Fig. 6C) suggests that medications might account for some of the observed effects on alpha power. Indeed, if one mentally combines the left half of Fig. 6B with the right half of Fig. 6C, it seems that the observed Healthy vs. DRFE alpha power differences can be almost entirely accounted for by recording system differences and AED effects; i.e. perhaps the difference between Healthy and PNES is driven by recording system differences, and the difference between PNES and DRFE is driven by medication effects in the majority of DRFE patients who were taking AEDs?

Thank you for this comment and we agree that as far is possible disentangling effects of medications and acquisition systems is important. We believe that the small sample size in the DRFE population without AEDs (N=5) added to this confusion. To increase the statistical power of this comparison, we performed a similar comparison in the PNES population (i.e., P(Normal) in PNES patients taking AEDs (N=27) vs not (N=44)) which is illustrated in Figure 6E. Although the effect of AEDs appears to be in the same direction (i.e., reduction in probability-of-normality), in contrast to the result in DRFE, the difference with respect to consumption of AEDs was not significant within the PNES patients (p=0.19).

Taken together, these concerns regarding potential confounding variables cast doubt on the validity of the authors' findings.

Finally, to further clarify whether the DRFE patients are truly different from PNES and/or healthy, we performed another comparison by homogenizing the PNES and DRFE populations. We selected subgroups who were taking AEDs within PNES (N=27) and DRFE (N=43) populations. It is important to note that these two populations share similar characteristics with respect to acquisition system, AEDs, and age-range (PNES age range: 19 – 62, DRFE age range: 18 – 66). Figure 6C illustrates the results of multiple comparisons performed on those probability-of-normality values between the two groups. We found that the difference between PNES and DRFE is still significant ($p<0.01$) when all confounders are controlled for. This finding firmly supports our main hypothesis that there are significant disruptions in the alpha-power characteristics of DRFE patients compared to subjects without epilepsy. To further support this result, we performed a classification experiment between PNES and DRFE, both patients taking AEDs, using the same framework described in Figure 2D. The results, illustrated in supplementary Figure 2 and Table 3, indicate an AUC of 0.62 and F1-scores above 70% in classifying those two groups. Based on these findings, we argue that the difference between healthy and DRFE is unlikely to be less significant.

Minor Concerns:

1. Introduction, line 12: "…in patients determined to have DRE"; might be better to rephrase as "in patients found to have DRE" to avoid double-meaning of the word 'determined'

Modified in the revised manuscript.

2. Introduction, "EEG is the primary method to diagnose epilepsy". Some readers might take umbrage with this statement, as epilepsy is a clinical diagnosis and History is usually the paramount diagnostic tool, particularly given the many limitations of EEG.

Thank you for this comment. We agree, and have corrected this statement.

3. Methods: Could the authors please specify the criteria by which certain channels were deemed to be outliers?

We apologize for the confusion. The outlier channels were annotated via visual inspection by the investigators of LEMON study. We did not remove any channels. This is now clarified in the manuscript.

4. Several figure legends refer to 'shaded areas' indicating confidence intervals, but these are not visible in the PDF I reviewed (unless the interval are extremely tight). Perhaps something got lost during file conversion?

Yes, we believe the shaded areas were lost during file conversion. We will work with the publishers to ensure this is addressed.

5. Figs. 3 and 4: I assume the head plots are top down views (i.e. not radiographic convention), but labeling L and R might help avoid confusion. Also, the authors state that CDF plots were "nearly symmetric" but some asymmetry is apparent in Fig. 3C—could the authors please comment?

We have added L and R to the headplots and corrected the statement to reflect the fact that the headplot is symmetric only in the low-alpha range.

6. The Discussion should include some comments on envisioned clinical applications of a test with an AUC of 0.8, including ways it could be helpful (i.e. avoid delayed diagnosis/treatment, as currently mentioned) and also unhelpful (i.e. morbidity of unnecessary testing/treatment when an imperfect biomarker is falsely positive).

We appreciate this suggestion, and have added a section in the discussion describing the potential clinical applications.

Thank you for the opportunity to review this interesting manuscript.

Thank you for the valuable feedback.

Reviewer: 2

The manuscript is well written, and all steps of the investigation can be easily followed. Nonetheless, I do have some reservations regarding the use of 'probability-of-normality' (PON). First of all, the abstract states that alpha power is reduced in DRFE etc, yet the manuscript only presents implicit evidence for this via the PON measure. The results would be much more tangible if the (averaged) alpha power fraction were used directly. One problem I see with the PON is that the necessary assumption that alpha power is reduced in epilepsy prevents to generalise the PON (e.g. a hypothetical disorder that shows an increase of alpha power would then show a higher PON than healthy controls). The use of the term PON can also be a bit confusing, as I first thought it refers to the likelihood that the log-transformed alpha power is normally distributed (Fig 2B). Additionally, the PON is roughly 0.45 in healthy controls; I would expect this to be close to 1.

Given the fact that the PON is based on monotone transformations (log, CDF) of the alpha power, and that the nonparametric tests (as well as the ROC) are rank based, I am convinced that the results would remain virtually unchanged if the alpha power between the test groups is compared directly. Otherwise, the use of an elaborate method such as the PON has to be better motivated, i.e. it has to be explained what it yields that alpha power doesn't.

Thank you for the positive feedback and the comments. We do acknowledge that the probability-of-normality (or PON) measure is custom defined for this work. However, this measure has broader applications going forward in the area of brain health and therefore of interest to us, and we chose to formalize the definition.

We agree that this approach implicitly assumes that diseases tend to decrease alpha power. However, this assumption is supported by neuroscience literature (Larsson and Kostov 2005, Aiyer, Novakovic et al. 2016, Knyazeva, Barzegaran et al. 2018, Abela, Pawley et al. 2019, Halgren, Ulbert et al. 2019).

The reason for average P(Normal) around 0.45 is that the measure uses the CDF function. The CDF takes a value of 0.5 for a normal distribution at the overall mean of the random variable. Although we did not make any assumption about the underlying distribution of the data, our data closely resembles a normal distribution (as evident from Figure 2B). Based on this model, someone with really high eyes closed alpha power will have P(Normal) approximately equal to1.

Furthermore, we also agree with the reviewer that no mathematical model is going to provide more information than what is available in the data. However, an approach to combine the individual feature-based differences within each individual (different brain regions and time windows) was necessary to develop an individual-level classification scheme and evaluate the clinical value of those differences. We have further clarified in this in our manuscript.

Minor points / typos:

We have fixed all the typos in the revised manuscript. Thank you for catching those.

Furthermore, we believe the shaded areas were lost and some text in the figures were squeezed during file conversion. We will work with the editorial office to fix this.

Reviewer: 3

Comments to the Author
This manuscript develops the recent interest in identification of features of apparently normal EEG that might identify the presence of a seizure disorder or epilepsy in patients. The translational potential of such approaches is high, due to the vast majority of clinically recorded EEGs in the epilepsy diagnosis pathway currently being viewed as normal and therefore clinically uninformative.

Thank you for this comment.

In general I think the findings presented in this manuscript are interesting, however I believe the authors could place their work in better context of the existing literature around interrogation of apparently normal EEG in the context of epilepsy, as well as consider some challenges relevant to group-level observations versus individual predictions, which are of importance for the ultimate translation of these findings. I expand on these comment below.

Thank you for this very useful feedback. We have included text in the revised manuscript to reflect your suggestions. We address specific concerns below.

In this manuscript the authors state that "we hypothesized that even normal EEGs might contain subtle electrophysiological cues of epilepsy". There is a substantial body of work that explores this and presents strong evidence for it, so I think it is not the best use of language to describe it as a hypothesis, particularly as it gives the implication that the authors themselves generated it, which is I am sure not the impression they sought to give. For example in the specific context of alpha power Larson et al Clin Neurophys 116, 2701 (2005) first identified a relationship between decrease in alpha power and people with epilepsy. Whilst they didn't focus specifically on DRFE vs well-controlled, it is a clear extension of this work that the hypothesis would be "lower frequency" correlates with poorer likelihood of seizure freedom. I feel the authors should include reference to this manuscript and a discussion of how their work builds on its findings.

Thank you for the comment and the reference. We agree with the reviewer's assessment that our work builds on several existing reports on alpha rhythm and epilepsy including (Larsson and Kostov 2005). However, our study makes several unique contributions: a) evaluation of alpha-rhythm abnormalities in DRFE using multiple EEG windows extracted from routine EEG recordings that were visually classified by epileptologists as normal; b) characterizing the spatial patterns of alpha-spectral abnormalities in DRFE and their value in identifying the seizure generating hemisphere; c) development of an approach to combine alpha-spectral abnormalities from several channels and windows to generate probability of normality measures; d) classification analysis to clearly evaluate the diagnostic value of alpha-spectral abnormalities; and e) validating our findings against confounders such as acquisition system/condition differences, age, and medications. We have included the reference and modified our introduction and discussion sections to reflect the reviewer's suggestion.

Now in their introduction, I feel it is important for the authors to give a more holistic overview of ongoing research that explores the relationship between apparently normal EEG and risk of epilepsy. For example, back in 2014 Chowdhury et al PLOS ONE 110136 demonstrated for the first time that network properties, as inferred from background EEG, were altered, both in people with generalized epilepsies as well as their first degree relatives. Of note to the present study is that the frequency band within which the EEG was filtered and these networks were inferred was termed "low alpha" defined as 6-9Hz. Building on this, the same group Schmidt et al 2014 PLOS Comp Biol 1003947 demonstrated that changes in these networks

supported a dynamic adaptation to hypersynchrony, making it easier for seizures to emerge. Most recently, Woldman et al 2020 Sci Rep 10 7043 used a computer model to demonstrate how altered functional networks in these low frequency bands could support EEG-like activity that was either confined to a single hemisphere, or bilaterally spread. This provided evidence for network alteration being an enduring feature of seizures, and that it is possible to identify a focal or generalized seizure phenotype from apparently normal background EEG. Further work of relevance to support the use of background EEG in the context of seizure/epilepsy identification is that of Verhoeven et al 2017 NeuroImage Clinical 17, 10-15 who presented an algorithm for identifying cases of temporal lobe epilepsy from background EEG in the absence of interictal spikes. This study used source-based network reconstruction across 80 or so regions of interest and achieved approaching 90% accuracy in a small cohort of 40 subjects. Very recently Pegg et al 2021 Epilepsia 62, 492 considered the difference between drug resistant and well controlled IGE cases, finding that properties of the inferred networks correlated strongly with seizure freedom or poor control. The authors may find a recent systematic review of graph measures in interictal data from IGE from the same group useful: Pegg et al 2020 Epilepsy Behav 107013.

Thank you for the extensive literature, and we appreciate this comment and the chance to better place this effort in the context of prior studies. We have added references to existing work looking at EEG connectivity measures to differentiate epilepsy and its subtypes from control groups using normal EEG segments. We believe that connectivity-based measures can add independent value and can be combined with univariate measures such as the alpha power to increase the diagnostic value of normal EEGs. Our future work will investigate this possibility.

In the methods, I could not understand the rationale for handedness to determine the "dominant" hemisphere, and why this would be important in terms of revealing the seizure generating propensity of the brain. For example, contrasting with the approach mentioned earlier of Woldman et al 2020? Could the authors expand a little as to why the dominant hemisphere is important, given there was a split of left and right sided epilepsies in the cohort?

We apologize for the confusion. Directly quoting from our introduction, "there is evidence that patients with epilepsy who have seizures originating from their dominant hemisphere can experience relatively more disruptions in their normal brain function, compared to those with seizure foci in their non-dominant hemispheres (Waldman, Camarillo-Rodriguez et al. 2018). Therefore, we further hypothesize that in addition to indicating the potential for epilepsy, alpha rhythm abnormalities can also help lateralize the seizure focus in focal epilepsy". This perspective derives from the central idea that normal brain function is disrupted in diseases such as epilepsy and even more so when seizure foci are in the dominant hemisphere. The physiology for this finding is not entirely clear, but may reflect that there are clearly functional differences between dominant and non-dominant hemispheres. Furthermore, we believe that this effect is distinct from focal differences in alpha power. Our study provides evidence for this idea using alpha power in right-handed DRFE patients (Figure 4D).

The results are in general clearly presented, but again I feel it would be helpful for the reader if the authors were to describe the key differences between their current approach, and that of Larson et al mentioned earlier.

Addressed above.

In their discussion, I feel the authors might like to give some consideration to potential limitations when it comes to the application of these findings at an individual level. Here I refer to Schmidt et al 2016 Epilepsia 57, e200. In this study the authors consider a cohort for people with IGE vs healthy controls and used a classifier to explore the performance of markers (including alpha power) previously identified as distinguishing between the cohorts at a group level. Alpha power (in the sense introduced by Larson) was

shown to have 0 accuracy at an individual level, and so would not be an appropriate marker. The authors may wish to discuss their findings in the context of group level effects vs individual differences.

Our results described in Table 2 and Figure 5 both were obtained at an individual subject level. Although we performed all our analyses using multiple 10-second windows within each subject, the window-level probability estimates were combined at an individual level to classify each subject to a group (healthy vs DRFE or the hemisphere generating seizures). Contrary to the findings of (Schmidt, Woldman et al. 2016), our results indicate that individual healthy and DRFE patients can be differentiated with substantial accuracy using the alpha power features obtained from normal EEGs (F1-score > 70%). We also note that (Schmidt, Woldman et al. 2016) utilized peak alpha power as opposed to the total alpha-band power we utilized.

Finally, in their discussion of limitations the authors rightly raise the issue that they have compared people with epilepsy with health controls. However they may wish to frame this more in the context of diagnostic/prognostic biomarker studies, where a phase I/II study would be to demonstrate that a marker is effective, and that it can distinguish those with a condition, from those clearly without the condition. For example Yee et al 2018 Bioanalysis 10, 4155.

Thank you for the suggestion. We have added text in the discussion to reflect this.

Editorial Office comments:

-Fig1b:please, use different line styles to identify the data groups.

Modified.

-Please supply a graphical abstract. A graphical abstract is a visual representation of the central finding or methodology of the paper. It will be displayed in the table of contents. Authors should strive to make the graphical abstract informative, interesting, visually appealing, and straightforward (no caption needed, no abbreviations except gene names). Please upload as a separate high resolution image file (tiff preferred). The technical requirements for the graphical abstracts are:
--size of the submitted image: 1200 pixels square at 300dpi
--font of the text: Arial 12-16 point (smaller fonts will not be legible online)

Thank you for the suggestion. We will provide a graphical abstract with the final version.

-Please add an Abbreviated Summary (up to 50 words) of your paper that captures the main purpose and conclusions of the work. The summary should state the main results written in the third person (‘[1st author surname] et al. report that …’) and conclusion. This summary will be used in the contents list online and should not contain any abbreviations other than those on the accepted abbreviations list and accepted gene/protein names, making it accessible to the non-specialist. Please upload it as a Word document. Allowed abbreviations: AIDS; ANOVA; AMPA; ATP; A,T,C,G; CNS; CSF; CT; DNA; ECG; EEG; EMG; GABA; HIV; MRI; NMDA; PET; PCR; RNA.

Thank you for the suggestion. We will provide an abbreviated summary with the final version.

- To comply with our ethical standards, the appropriate checklist must be included (see Reporting Guideline section within 'Instructions to authors').

References

Abela, E., A. D. Pawley, C. Tangwiriyasakul, S. N. Yaakub, F. A. Chowdhury, R. D. C. Elwes, F. Brunnhuber and M. P. Richardson (2019). "Slower alpha rhythm associates with poorer seizure control in epilepsy." Ann Clin Transl Neurol **6**(2): 333-343.

Aiyer, R., V. Novakovic and R. L. Barkin (2016). "A systematic review on the impact of psychotropic drugs on electroencephalogram waveforms in psychiatry." Postgrad Med **128**(7): 656-664.

DiGirolamo, G. J., N. Patel and C. L. Blaukopf (2016). "Arousal facilitates involuntary eye movements." Exp Brain Res **234**(7): 1967-1976.

Halgren, M., I. Ulbert, H. Bastuji, D. Fabo, L. Eross, M. Rey, O. Devinsky, W. K. Doyle, R. Mak-McCully, E. Halgren, L. Wittner, P. Chauvel, G. Heit, E. Eskandar, A. Mandell and S. S. Cash (2019). "The generation and propagation of the human alpha rhythm." Proc Natl Acad Sci U S A **116**(47): 23772-23782.

Jung, T. P., S. Makeig, M. Stensmo and T. J. Sejnowski (1997). "Estimating alertness from the EEG power spectrum." IEEE Trans Biomed Eng **44**(1): 60-69.

Kaiser, A. K., M. T. Gnjezda, S. Knasmuller and W. Aichhorn (2018). "Electroencephalogram alpha asymmetry in patients with depressive disorders: current perspectives." Neuropsychiatr Dis Treat **14**: 1493-1504.

Kleifges, K., N. Bigdely-Shamlo, S. E. Kerick and K. A. Robbins (2017). "BLINKER: Automated Extraction of Ocular Indices from EEG Enabling Large-Scale Analysis." Front Neurosci **11**: 12.

Knyazeva, M. G., E. Barzegaran, V. Y. Vildavski and J. F. Demonet (2018). "Aging of human alpha rhythm." Neurobiol Aging **69**: 261-273.

Larsson, P. G. and H. Kostov (2005). "Lower frequency variability in the alpha activity in EEG among patients with epilepsy." Clin Neurophysiol **116**(11): 2701-2706.

Liu, X. Y., J. Wu and Z. H. Zhou (2009). "Exploratory undersampling for class-imbalance learning." IEEE Trans Syst Man Cybern B Cybern **39**(2): 539-550.

Newson, J. J. and T. C. Thiagarajan (2018). "EEG Frequency Bands in Psychiatric Disorders: A Review of Resting State Studies." Front Hum Neurosci **12**: 521.

Schmidt, H., W. Woldman, M. Goodfellow, F. A. Chowdhury, M. Koutroumanidis, S. Jewell, M. P. Richardson and J. R. Terry (2016). "A computational biomarker of idiopathic generalized epilepsy from resting state EEG." Epilepsia **57**(10): e200-e204.

Waldman, Z. J., L. Camarillo-Rodriguez, I. Chervenova, B. Berry, S. Shimamoto, B. Elahian, M. Kucewicz, C. Ganne, X. S. He, L. A. Davis, J. Stein, S. Das, R. Gorniak, A. D. Sharan, R. Gross, C. S. Inman, B. C. Lega, K. Zaghloul, B. C. Jobst, K. A. Davis, P. Wanda, M. Khadjevand, J. Tracy, D. S. Rizzuto, G. Worrell, M. Sperling and S. A. Weiss (2018). "Ripple oscillations in the left temporal neocortex are associated with impaired verbal episodic memory encoding." Epilepsy Behav **88**: 33-40.
